# The caregiving role influences Suboptimal Health Status and psychological symptoms in unpaid carers

**DOI:** 10.1007/s13167-024-00370-8

**Published:** 2024-07-31

**Authors:** Monique Garcia, Zheng Guo, Yulu Zheng, Zhiyuan Wu, Ethan Visser, Lois Balmer, Wei Wang

**Affiliations:** 1 Center for Precision Health, School of Medical and Health Science, Edith Cowan University, Perth, WA 6027 Australia; 2 School of Medical and Health Science, Edith Cowan University, Perth, WA 6027 Australia; 3 The First Affiliated Hospital of Shantou University Medical College, Shantou, 515041 Guangdong China

**Keywords:** Suboptimal Health Status, Predictive preventive personalised medicine (PPPM / 3PM), Psychological symptoms, Carers, Suboptimal Health Status-25, Health risk assessment, Depression Anxiety Stress Scale-21

## Abstract

**Background:**

Suboptimal Health Status (SHS) is the physical state between health and disease. This study aimed to fill in the knowledge gap by investigating the prevalence of SHS and psychological symptoms among unpaid carers and to identify SHS-risk factors from the perspective of predictive, preventive and personalised medicine (PPPM).

**Methods:**

A cross-sectional study was conducted among 368 participants who were enrolled from Australia, including 203 unpaid carers as cases and 165 individuals from the general population as controls. SHS scores were measured using SHSQ-25 (Suboptimal Health Status Questionnaire-25), whilst psychological symptoms were measured by DASS-21 (Depression, Anxiety and Stress Scale-21). Chi-square was used to measure SHS and psychological symptom prevalence. Spearman correlation analysis was utilised to identify the relationship between SHSQ-25 and DASS-21 scores. Logistic regression analysis was used for multivariate analysis.

**Results:**

The prevalence of SHS in carers was 43.0% (98/203), significantly higher than the prevalence 12.7% (21/165) in the general population (*p* < 0.001). In addition, suboptimal health prevalence was higher in female carers (50.3%; 95/189) than females in the general population (12.4%; 18/145). Logistic regression showed that the caregiving role influenced SHS, with carers 6.4 times more likely to suffer from SHS than their non-caring counterparts (aOR = 6.400, 95% CI = 3.751–10.919).

**Conclusions:**

Unpaid carers in Australia have a significantly higher prevalence of SHS than that in the general population and experience poorer health. The SHSQ-25 is a powerful tool that can be utilised to screen at-risk individuals to predict their risk of chronic disease development, an essential pillar for shifting the paradigm change from reactive medicine to that of predictive, preventive and personalised medicine (PPPM).

**Supplementary Information:**

The online version contains supplementary material available at 10.1007/s13167-024-00370-8.

## Background

The definition of “health” according to the World Health Organisation (WHO) is “a state of complete physical, mental, and social well-being and not merely the absence of disease or infirmity” [1]. Suboptimal Health Status (SHS) is the physical state between health and disease, characterised by the self-reporting of general malaise and ambiguous health complaints in the absence of a clinically diagnosable condition [2]. SHS is considered a “subclinical, reversible stage of pre-chronic disease” [2]. Chronic diseases are serious, global threats to human health, defined as diseases of long duration, slow progression, and are the major cause of adult morbidity and mortality worldwide [3]. Chronic diseases are responsible for 71% of global deaths and kill more than 41 million people each year [1].

Total disease burden is measured by the metric “disability-adjusted life years” (DALY) [4, 5]. In Australia, there was a total of 5.5 million DALY lost, attributed to living with illness (52%) and premature death (48%), with chronic diseases causing the majority of the burden in year 2022 [5]. The impact of chronic diseases, however, extends far beyond healthcare to that of economies. A recent economic report measuring global “value of lost output” showed that the total lost output from the four major chronic diseases (cardiovascular disease (CVD), chronic respiratory disease, cancer, type two diabetes mellitus (T2DM)), plus mental disorders, from 2011 to 2030 to be approximately US$ 47 trillion [6]. For context, the entire economic output for Europe in 2016 was $17.8 trillion [6].

Predictive, preventive and personalised medicine (PPPM) shifts the paradigm from reactive healthcare to proactive healthcare, going beyond the state of the art by hypothesising that chronic diseases can be predicted—and even prevented—avoiding disease manifest altogether. Under PPPM’s working hypothesis, practitioners are offered a unique window of opportunity to employ targeted chronic disease prevention and treatment modalities [7]. Preventive actions, via personalised treatment algorithms, can then be prescribed. Identifying persons at risk of developing chronic diseases is an outcome shared by both PPPM and SHS [3, 8]. Assessment of SHS, via the Suboptimal Health Status Questionnaire-25 (SHSQ-25), has demonstrated the ability to predict the risk of certain chronic diseases, such as CVD [9], and is currently the most widely utilised questionnaires in accordance with PPPM.

Effective PPPM approaches, such as SHS, have the capacity to significantly mitigate the impact of chronic diseases on healthcare systems and society as a whole [10]. These potential benefits have the capacity to extend to improvements in quality-of-life outcomes for entire populations and state-of-the-art healthcare delivery by clinicians. The criticality of investing in such measures was highlighted by WHO recent calls for evidence-based, cost-effective disease prevention strategies to alleviate the burgeoning costs and complications of chronic diseases [4]. SHS, and its role in reducing the prevalence of chronic diseases on a global scale, warrants special attention from the perspective of PPPM.

SHS was first identified as a public health challenge in China, with its prevalence expected to escalate worldwide [2]. On behalf of the Suboptimal Health Study Consortium (SHSC), a multi-centre international research group dedicated to SHS from the perspectives of PPPM, the suboptimal health status questionnaire-25 (SHSQ-25) has been established as a reliable, subjective health measure for the detection of SHS [11]. The SHSQ-25 has been validated and translated into numerous major ethnic groups including African, Asian, Caucasian, Korean, Arabic, and Persian [3, 10, 12–18]. Using the SHSQ-25, an increasing number of studies have demonstrated that SHS is associated with multiple chronic diseases, such as type II diabetes mellitus, CVD and mental health conditions [8]. Stress has been identified as a key contributing factor of SHS [2]. With our increasingly chaotic lifestyles, societal changes, social media use and work pressures, the stress-related disease epidemic has become an important public health issue [2]. Psychological symptoms are assessed as part of the SHSQ-25 [2]. Previous studies have found chronic psychosocial stress to be associated with SHS, with plasma cortisol levels significantly higher among people with SHS [19]. Another study found that SHS in Chinese college students was associated with psychological stress [20]. Considering that mental health disorders are the leading cause of DALYs globally, attributing to 37% of healthy life years lost from chronic diseases, the screening and detection of SHS in vulnerable and disadvantaged populations is paramount and should be a priority [21].

Informal, unpaid carers are one such group known to have unmet care needs, including mental health and stress disorders, compared to the general population [22, 23]. As defined by the International Alliance of Carer Organisations (IACO), a coalition of national carer organisations, a carer is “an unpaid individual, such as a family member, neighbour, friend or other significant individual, who takes on a caring role to support someone with a diminishing physical ability, a debilitating cognitive condition or a chronic life-limiting illness” [24]. Caring is an international issue, recognised in 2012 by the establishment of the IACO [24]. In Australia, there are approximately 2.65 million carers, accounting for 12.8% of the population [25, 26]. This figure is similar or higher in the UK, Europe and the USA [27]. The total replacement cost, defined as the total value that would be needed to replace the services provided by informal carers, has been estimated to be $77.9 billion a year in 2020 [28].

Despite mental health promotion efforts from governments, mental health conditions of unpaid carers have worsened since COVID-19, escalating the early development of chronic disease [23]. The impact of lockdowns and decreased access to care contributed to “an unprecedented public mental health crisis” [29].

From the perspective of PPPM, information pertaining to the health of unpaid carers (mental and physical) versus the general population is urgently needed for predictive diagnosis and targeted prevention [30]. This is especially important considering detection of SHS in carers has not yet been explored.

We conducted this study to estimate the prevalence rates of SHS and psychological symptoms among two populations: informal, unpaid carers and the general population (to act as the SHS reference point) to understand how the caring role influences SHS and psychological symptoms. We also aimed to quantify the correlation between SHS and psychological symptoms under the concept of PPPM. We propose that using the SHSQ-25, in the spirit of PPPM’s working hypothesis, this knowledge gap is addressed assessment of carers contributes to the paradigm shift from reactive medicine to PPPM medicine, going beyond the state of the art by predicting, preventing and personalising the treatments of chronic diseases in unpaid carers, ultimately transforming healthcare by improving the quality of life in carers and society as a whole.

## Methods

### Study participants

A community-based, association study was carried out from May 2021 to December 2022. The study was approved by Edith Cowan University Human Research Ethics Committee (HREC). Two populations were examined in this study, informal, unpaid carers as cases and the general population as controls. For carers, we purposively selected participants from community and not-for-profit organisations that were carer-specific, such as Carers Australia. For the general population, we purposively selected Australian Facebook groups whose members were from the general population. To promote cultural inclusion and diversity, we included all ethnic specific groups. Snowball sampling was utilised.

**Fig. 1:**
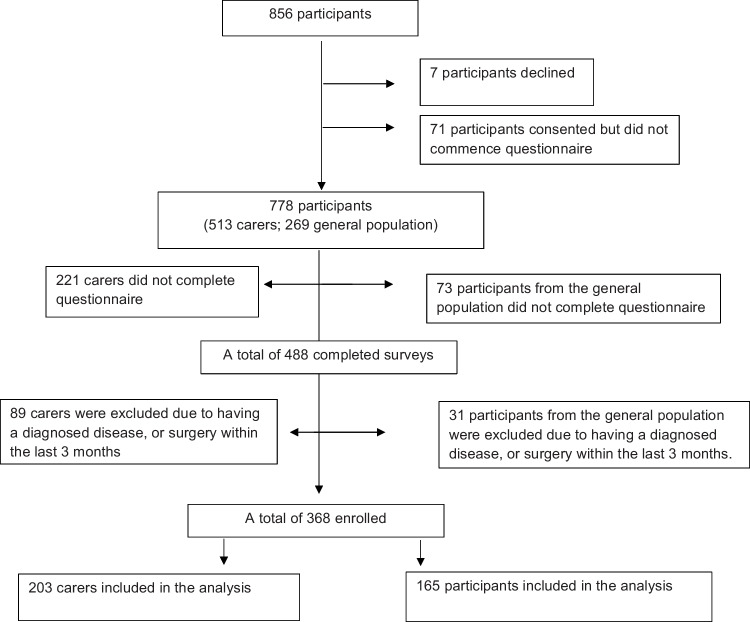
Flowchart of participants

A total of 856 participants were recruited for the study, with 78 participants choosing to not remain in the study (7 participants declined to participate; 71 participants consented but did not commence the survey) leaving 778 participants (513 carers; 269 members of the general population). The flowchart of participant enrolment is shown in Fig. 1.

The inclusion criteria for carers were (1) people who provided informal, unpaid care for a family member or friend, (2) no history or diagnosis of somatic diseases, (3) no history of diagnosis of psychiatric abnormalities, (4) no history or diagnosis of any disability and (5) no surgeries in the past 3 months. Carers who received income supplements from Centrelink for their caring role (such as carers’ payment or carers’ allowance) were considered unpaid carers and were eligible to participate in this study. The inclusion criteria for the general population were (1) people who had never provided informal, unpaid care for a family member or friend, (2) no history or diagnosis of somatic diseases, (3) no history of diagnosis of psychiatric abnormalities, (4) no history or diagnosis of any disability and (5) no surgeries in the past 3 months.

Among 856 participants, 310 carers (221 did not complete the questionnaire; 89 had a diagnosed disease) and 104 members of the general population (73 did not complete the questionnaire; 31 had a diagnosed disease) were also excluded. The self-administered, online surveys using Qualtrics XM software were disseminated to all participants to measure SHS and psychological symptoms of 368 individuals (203 carers; 165 members of the general population). Informed consent was gained electronically at the commencement of each survey by clicking the button option of “I consent to this study”. Participants then clicked the button “next” to begin the survey. The survey was completed by the participants using their own electronic devices (such as a mobile phone or tablet). The survey took approximately 20–25 min. Each participant was de-identified to protect his or her privacy.

### SHS evaluation

SHS was measured using the SHSQ-25, a robust, reliable, non-invasive and cost-effective screening tool for the detection of SHS [2]. The SHSQ-25 includes 25 items and assesses health across 5 domains [2]. These include fatigue, mental status, cardiovascular system, digestive tract and the immune system [2]. Participants were asked to rate each item on a 5-point Likert-type style, based on how often they have felt that way over the last 3 months. Raw scores of 1 to 5 on the questionnaire were recoded as 0–4. An individual’s total suboptimal health score was calculated by adding the ratings from the 25 items together [2]. As this study marked the first time that SHS has been evaluated in an Australian population, it was necessary to establish the cut-off value for SHS in this current study. As described elsewhere, the method of establishing the cut-off value for SHS is determined using a one-tailed 90% upper limit of SHSQ-25 scores [9]. Using this approach, we determined the SHS cut-off value for an Australian population was SHS score of 39. Participants with scores of 39 or greater are considered to have SHS, whilst scores of less than 39 are considered to have optimal health (Table 1).

**Table 1:** Suboptimal Health Questionnaire-25 (SHSQ-25); these questions ask about health experiences occurring within the last three months. Each question needs an “x” marked in the appropriate box, with scores totalled for a SHS score

In the preceding 3 months, how often was it that you (your)…	1	2	3	4	5	Score
	Never or almost never	Occasionally	Often	Very often	Always	
1. Were exhausted without greatly increasing your physical activity?						
2. Experienced fatigue that could not be substantially alleviated by rest?						
3. Were lethargic when working?						
4. Suffered from headaches?						
5. Suffered from dizziness?						
6. Eyes ached or were tired?						
7. Suffered from a sore throat?						
8. Muscles or joints felt stiff?						
9. Have pain in your shoulder/neck/waist?						
10. Have a heavy feeling in your legs when walking?						
11. Felt out of breath while sitting still?						
12. Suffered from a chest congestion?						
13. Were bothered by heart palpitations?						
14. Appetite was poor?						
15. Suffered from heartburn?						
16. Suffered from nausea?						
17. Could not tolerate cold environments?						
18. Had difficulty falling asleep?						
19. Had trouble with waking up during night? i.e., kept waking up at night						
20. Had trouble with your short-term memory?						
21. Could not respond quickly?						
22. Had difficulty concentrating?						
23. Were distracted for no reason?						
24. Felt nervous of jittery?						
25. Caught a cold in the past 3 months?						
Total						

### Psychological symptom evaluation

The psychological symptoms of participants were assessed by the Depression, Anxiety and Stress Scale (DASS-21) [31]. The DASS-21 is a self-reporting questionnaire comprising a total of 21 items. Within the DASS-21 are three separate scales containing 7 items each. These scales measure the emotional states of depression, anxiety and stress, respectively [31]. The depression scale measures dysphoria, hopelessness, devaluation of life, self-deprecation, lack of interest, anhedonia and inertia; the anxiety scale measures autonomic arousal, skeletal muscle effects, situational anxiety and subjective experience of anxious affect; the stress scale measures difficulty relaxing, nervous arousal, impatience and becoming easily upset, irritable or over-reactive [31]. Participants were asked to rate each statement on a 4-point Likert-type scale, based on how much the statement applied to them over the last week. An individual’s score for each scale (depression, anxiety and stress) was calculated by summing the ratings of each scale’s 7 items and then multiplied by 2. The cut-off scores for the depression scale are normal (0–9), mild (10–13), moderate (14–20), severe (21–27) and extremely severe (28+); the anxiety scale is normal (0–7), mild (8–9), moderate (10–14), severe (15–19) and extremely severe (20+); the stress scale is normal (0–14), mild (15–18), moderate (19–25), severe (26–33) and extremely severe (34+) [24].

### Statistical analysis

The statistical analysis was performed by SPSS (version 27.0, IBM, New York, USA). The normality of the data was performed using the Kolmogorov test. Data were expressed as frequency and percentages for categorical variables. Parametric continuous variables were expressed as mean and ±SD, with non-parametric continuous variables expressed as median and interquartile ranges (IQR) (P_25_~P_75_). A Pearson chi-squared test (*X*^2^) was used to test the association between the proportion of variables with suboptimal health and optimal health status, as well as the proportion of variables with psychological symptoms including depression, anxiety and stress, respectively, and no psychological symptoms. A Mann-Whitney test or a Kruskal-Wallis test was used to compare the median between groups. Since neither SHS nor psychological symptoms did not meet the assumption for Pearson’s correlation, the Spearman rho correlation was used to analyse the relationship between SHSQ-25 scores and DASS-21 scores, respectively. A multivariate logistic regression model was performed to test the association between SHS and psychological symptoms, by which the adjusted odds ratio (aOR) and 95% confidence intervals (CI) were calculated. A *p* value < 0.05 was considered statistically significant.

## Results

### Characteristics of participants

The characteristics of the participants enrolled in the study had an average age of 45 years (quartile 37~51, range 18~75) and a gender ratio of 334/32/2 (female/male/non-binary). Of the total 368 participants, 203 (55.2%) were carers, and 165 (44.8%) were from the general population. The average age of carers was 46 years (quartile 40–53, range 21–75) compared with 40 years (quartile 32–49, range 18–72) for the general population. The gender ratio of carers was 189/13/1 (female/male/non-binary), whereas the gender ratio of the general population was 145/19/1 (female/male/non-binary). A total of 93.1% of carers were female compared to 87.9% of the general population; 6.4% of carers were male compared to 11.5% of the general population, and 0.5% of carers were non-binary compared to 0.6% of the general population. Among the total 203 carers, 160 (78.8%) lived in metro areas compared to 144 (87.3%) of the general population; 42 (20.7%) of carers lived in rural areas compared to 18 (10.9%) of the general population; and 1 (0.5%) carer lived in remote areas of Australia compared to 3 (1.8%) members of the general population.

### Prevalence of SHS

As shown in Table 2, the prevalence of SHS was significantly higher in carers than that in the general population (43.0% vs 12.7%), indicating carers are at significantly higher risk of SHS compared to the general population. This was further explored, and it was identified that female carers, aged between 25 and 44 years, were at significantly higher risk of SHS than that in the general population (50.3% vs 12.4%, 50.7% vs 13.5%, respectively). As shown in Table 2, carers were more susceptible to SHS than the general population despite having the same educational attainment, annual income or marital status. This was also observed for housing and living situations, whether individuals lived in metro/rural regions, whether individuals had a pension/healthcare card or not, private health insurance or not, or dependent children or not. This indicates that irrespective of demographics, carers are at significantly greater risk of SHS than the general population.

**Table 2:** Characteristics of participants

Variables			Total (*N* = 368)	Healthy (%) (*N* = 249)	SHS (%) (*N* = 119)	*X*^2^	*p* value
Gender	Male	General population	19	17 (89.5)	2 (10.5)	0.167	0.683
		Carer	13	11 (84.6)	2 (15.4)		
	Female	General population	145	127 (87.6)	18 (12.4)	52.512	***<0.001***
		Carer	189	94 (49.7)	95 (50.3)		
	Non-binary	General population	1	0 (0.0)	1 (100.0)	+	+
		Carer	1	0 (0.0)	1 (100.0)	+	+
Education	Primary school	General population	1	1 (100.0)	0 (0.0)	+	+
		Carer	2	2 (100.0)	0 (0.0)	+	+
	High school	General population	25	17 (68.0)	8 (32.0)	0.110	0.740
		Carer	36	23 (63.9)	13 (36.1)		
	TAFE/trade	General population	46	40 (87.0)	6 (13.0)	39.645	***<0.001***
		Carer	75	21 (28.0)	54 (72.0)		
	Bachelor’s degree	General population	54	48 (88.9)	6 (11.1)	9.739	***0.002***
		Carer	64	41 (64.1)	23 (35.9)		
	Postgraduate degree	General population	39	38 (97.4)	1 (2.6)	10.403	***0.001***
		Carer	26	18 (69.2)	8 (30.8)		
Income	Under $50k	General population	23	17 (73.9)	6 (26.1)	6.567	***0.010***
		Carer	76	33 (43.4)	43 (56.6)		
	$50–69k	General population	28	22 (78.6)	6 (21.4)	5.119	***0.024***
		Carer	30	15 (50.0)	15 (50.0)		
	$70–89k	General population	27	25 (92.6)	2 (7.4)	7.327	***0.007***
		Carer	32	20 (62.5)	12 (37.5)		
	$90–109k	General population	22	22 (100.0)	0 (0.0)	12.298	***<0.001***
		Carer	23	13 (56.5)	10 (43.5)		
	Over $110k	General population	65	58 (89.2)	7 (10.8)	14.672	***<0.001***
		Carer	42	24 (57.1)	18 (42.9)		
Marital status	Never married	General population	57	48 (84.2)	9 (15.8)	11.874	***0.001***
		Carer	32	16 (50.0)	16 (50.0)		
	Married	General population	79	72 (91.1)	7 (8.9)	25.388	***<0.001***
		Carer	130	76 (58.5)	54 (41.5)		
	Widowed	General population	5	4 (80.0)	1 (20.0)	1.742	0.464
		Carer	3	1 (33.3)	2 (66.7)		
	Divorced/separated	General population	24	20 (83.3)	4 (16.7)	15.776	***<0.001***
		Carer	38	12 (31.6)	26 (68.4)		
Locality	Metro	General population	144	125 (86.8)	19 (13.2)	49.474	***<0.001***
		Carer	160	78 (48.8)	82 (51.2)		
	Rural	General population	18	16 (88.9)	2 (11.1)	4.369	***0.037***
		Carer	42	26 (61.9)	16 (38.1)		
	Remote	General population	3	3 (100.0)	0 (0.0)	+	+
		Carer	1	1 (100.0)	0 (0.0)	+	+
Age	18–24 years	General population	16	12 (75.0)	4 (25.0)	0.643	0.423
		Carer	2	2 (100.0)	0 (0.0)		
	25–44 years	General population	89	77 (86.5)	12 (13.5)	26.308	***<0.001***
		Carer	73	36 (49.3)	37 (50.7)		
	45–64 years	General population	58	53 (91.4)	5 (8.6)	27.915	***<0.001***
		Carer	120	61 (50.8)	59 (49.2)		
	65 years and over	General population	2	2 (100.0)	0 (0.0)	0.625	0.429
		Carer	8	6 (75.0)	2 (25.0)		
Housing	Renting from real estate	General population	59	52 (88.1)	7 (11.9)	28.952	***<0.001***
		Carer	53	21 (39.6)	32 (60.4)		
	Public housing	General population	3	2 (66.7)	1 (33.3)	0.444	0.505
		Carer	9	4 (44.4)	5 (55.6)		
	Being paid off	General population	82	70 (85.4)	12 (14.6)	21.973	***<0.001***
		Carer	87	45 (51.7)	42 (48.3)		
	Fully owned	General population	21	20 (95.2)	1 (4.8)	7.156	***0.007***
		Carer	54	35 (64.8)	19 (35.2)		
Living situation	Living alone	General population	29	23 (79.3)	6 (20.7)	12.069	***0.001***
		Carer	27	9 (33.3)	18 (66.7)		
	Living with my partner	General population	103	95 (92.2)	8 (7.8)	31.906	***<0.001***
		Carer	134	80 (59.7)	54 (40.3)		
	Living with my parents	General population	12	9 (75.0)	3 (25.0)	2.217	0.239
		Carer	15	7 (46.7)	8 (53.3)		
	Living with other family/friends	General population	21	17 (81.0)	4 (19.0)	10.789	***0.001***
		Carer	27	9 (33.3)	18 (66.7)		
Pension card	Yes	General population	26	21 (80.8)	5 (19.2)	12.700	***<0.001***
		Carer	101	42 (41.6)	59 (58.4)		
	No	General population	139	123 (88.5)	16 (11.5)	23.855	***<0.001***
		Carer	102	63 (61.8)	39 (38.2)		
Private health	Yes	General population	108	98 (90.7)	10 (9.3)	26.355	***<0.001***
		Carer	110	67 (60.9)	43 (39.1)		
	No	General population	57	46 (80.7)	11 (19.3)	22.767	***<0.001***
		Carer	93	38 (40.9)	55 (59.1)		
Dependents	Yes	General population	93	82 (88.2)	11 (11.8)	41.482	***<0.001***
		Carer	117	53 (45.3)	64 (54.7)		
	No	General population	72	62 (86.1)	10 (13.9)	12.828	***<0.001***
		Carer	86	52 (60.5)	34 (39.5)		

+No statistics computed because SHS status is a constant

We also explored lifestyle/behaviour factors to understand whether they influenced the prevalence of SHS. As shown in Tables S1-S4, we found the prevalence of SHS was significantly higher in carers than in the general population irrespective of lifestyle/behaviour factors. For example, the prevalence of SHS in carers who led a sedentary lifestyle and spent most of their day sitting was significantly higher compared to the general population who did the same (46.1% vs 13.2%) (Table S1); carers who met with family member/s once every couple of months had a higher prevalence of SHS than those in the general population (64.7% vs 11.8%) (Table S2); carers who ate no serves of fruit per day were more susceptible to SHS compared to the general population (62.2% vs 31.8%) (Table S3). Carers who did not participate in any mild/moderate exercise were more susceptible to SHS than the general population (54.3% vs 8.1%) (Table S4). These findings indicate the caring role influences SHS.

### Total SHS scores and domains

As shown in Table 3, carers had significantly higher domain scores and IQR compared to that of the general population. For example, fatigue scores in carers were 17.0 (IQR 13.0~22.0) vs 9.0 (IQR 6.0~14.0) for the general population, cardiovascular scores were 1.0 (IQR 0.0~3.0) vs 1.0 (IQR 0.0~1.0), digestive scores were 3.0 (IQR 1.0~4.0) vs 1.0 (IQR 0.0–3.0), immune scores were 2.0 (IQR 1.0~4.0) vs 2.0 (IQR 1.0~3.0), and mental health scores were 12.0 (IQR 9.0~17.0) vs 6.0 (IQR 3.0~11.0). The median summed total SHS scores and IQRs were significantly higher in carers than that in the general population (38.0, IQR 28.0~48.0 vs 20.0, IQR 13.0–32.0). This indicates carers suffered poorer overall health than that in the general population, with the differentiator being the caring role.

**Table 3:** Scores from the SHSQ-25 for participants within respective populations

Variables	*n*	Total score	Mental	Dimensions of the SHSQ-25			
				Fatigue	Cardiovascular	Digestive	Immune
General population	165	20.0 (13.0–32.0)	6.0 (3.0–11.0)	9.0 (6.0–14.0)	1.0 (0.0–1.0)	1.0 (0.0–3.0)	2.0 (1.0–3.0)
Carer	203	38.0 (28.0–48.0)*	12.0 (9.0–17.0)*	17.0 (13.0–22.0)	1.0 (0.0–3.0)*	3.0 (1.0–4.0)*	2.0 (1.0–4.0)^+^

*p < 0.01 for statistical tests

^+^*p* = 0.01 for statistical tests

Irrespective of demographic factors, carers had higher total SHS scores than that in the general population (Table S5). For example, the median total SHS score was higher among female carers (39.0, IQR 29.0~48.5) than females in the general population (20.0, IQR 13.0~32.0) indicating females who provide care are akin to suffering poorer health. Carers aged 25–44 had a median total SHS score of 39.0 (IQR 29.0–48.5), whereas the general population aged 25–44 had a median total SHS score of 19.0 (IQR 13.0–32.5). This trend was also observed for marital status, education level, income, housing and living situation, whether individuals had a pension/healthcare card or not, private health insurance or not, or dependent children or not.

### Psychological symptoms of participants

As shown in Table 4, the prevalence of depressive symptoms in carers is significantly higher than that in the general population (75.4% vs 32.1%). This was also observed for the prevalence of anxiety symptoms (69.0% vs 31.1%) and stress symptoms (72.9% vs 33.9%), respectively. This indicates carers are more susceptible to depression, anxiety and stress symptoms than the general population and suffer from more than twice the number of psychological symptoms.

**Table 4:** Prevalence of depression, anxiety and stress symptoms

Variable	Population	*n*	Symptoms (%)	No symptoms (%)	*X*^2^	*p* value
Depression	General population	165	53 (32.1)	112 (67.9)	69.085	<0.001
	Carer	203	153 (75.4)	50 (24.6)		
Anxiety	General population	165	52 (31.15)	113 (68.5)	51.159	<0.001
	Carer	203	140 (69.0)	63 (31.0)		
Stress	General population	165	56 (33.9)	109 (66.1)	55.943	<0.001
	Carer	203	148 (72.9)	55 (27.1)		

DASS-21 scores were all statistically higher among carers than those in the general population. As shown in Table 5, the median depression score and IQR in carers are 16.00 (10.00–28.00) compared to 4.00 (2.00–12.00) in the general population. Similarly, the median anxiety score and IQR in carers are 10.00 (6.00–18.00) compared to 4.00 (0.00–8.00) in the general population, and the median stress score and IQR in carers are 22.00 (14.00–28.00) compared to 12.00 (6.00–18.00). These scores were all found to be statistically significant (*p* < 0.001).

**Table 5:** Scores of DASS-21 by domains

Population	*n*	Depression score	Anxiety score	Stress score
General population	165	4.0 (2.0–12.0)	4.0 (0.0–8.0)	12.0 (6.0–18.0)
Carers	203	16.0 (10.0–28.0)*	10.0 (6.0–18.0)*	22.0 (14.0–28.0)*

*DASS-21* Depression, Anxiety, Stress Scale-21

Data of continuous variables expressed as medians and interquartile ranges (P_25_~P_75_)

*p < 0.01 for statistical tests

As shown in Table S6, carers have higher DASS-21 scores than those in the general population, irrespective of demographic factors. For example, depression scores were higher in female carers than females in the general population (18.0; IQR 10.0~28.0 vs 4.0; IQR 2.0~12.0) (*p* < 0.001) and higher in male carers than males in the general population (16.0; IQR 8.0~18.0 vs 4.0; IQR 0.0~16.0) (*p* 0.018), indicating carers suffered more depression than the general population. Anxiety scores among carers who lived in metro areas (12.0, IQR 6.0–18.0) were higher than the general population who lived in metro areas (4.0, IQR 0.0–8.0) (*p* < 0.001). Carers who rented privately had higher stress scores (24.0, IQR 16.0–31.0) than the general population who rented privately (12.0; IQR 6.0–22.0) (*p* < 0.001). This trend was also observed for marital status, education level, income, housing and living situation, whether individuals had a pension/healthcare card or not, private health insurance or not, or dependent children or not.

### SHS and psychological symptoms

**Fig. 2:**
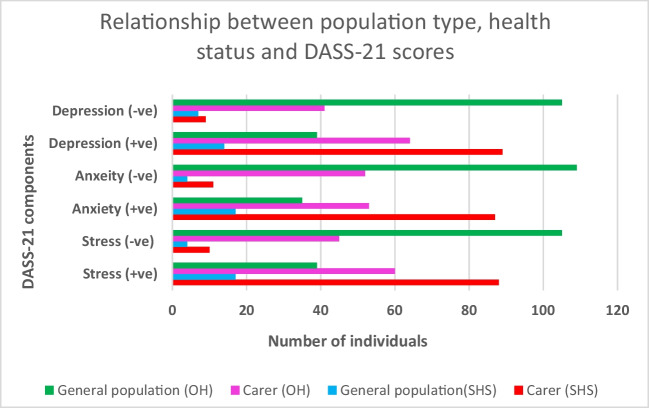
Frequencies of participants with psychological symptoms (DASS-21). +, positive for relevant symptom; −, negative for relative symptom

As shown in Table 6, the prevalence of depressive symptoms among SHS carers is significantly higher than that in the SHS general population (58.2% vs 26.4%) (*p* < 0.001). This was also observed for the prevalence of anxiety symptoms among SHS carers compared to the SHS general population (62.1% vs 32.7%) (*p* < 0.001) and stress symptoms among SHS carers compared to the SHS general population (59.5% vs 30.4%) (*p* < 0.001). SHS carers had significantly higher depression scores than the general population group (22.0; IQR 14.0~30.5 vs 12.0; IQR 6.0~25.0) (*p* 0.013). This was also observed in healthy carers, whose DASS-21 scores were significantly higher than the healthy general population group, respectively (14.0; IQR 4.0~22.0 vs 4.0; IQR 2.0~10.0) (*p* < 0.001) (8.0; IQR 4.0~12.0 vs 2.0; IQR 0.0~6.0) (*p* < 0.001) (16.0; IQR 12.00~25.00 vs 10.00; IQR 4.00~16.00) (*p* < 0.001) (Fig. 2).

**Table 6:** DASS symptom prevalence and SHS

Variables		Population	Total	Healthy (%)	SHS (%)	*p* value
Depressive symptoms	Yes	General populations	53	39 (73.6)	14 (26.4)	***<0.001***
		Carer	153	64 (41.8)	89 (58.2)	
	No	General populations	112	105 (93.8)	7 (6.3)	
		Carer	50	41 (82.0)	9 (18.0)	
Anxiety symptoms	Yes	General populations	52	35 (67.3)	17 (32.7)	***<0.001***
		Carer	140	53 (37.9)	87 (62.1)	
	No	General populations	113	109 (96.5)	4 (3.5)	
		Carer	63	52 (82.5)	11 (17.5)	
Stress symptoms	Yes	General populations	56	39 (69.6)	17 (30.4)	***<0.001***
		Carer	148	60 (40.5)	88 (59.5)	
	No	General populations	109	105 (96.3)	4 (3.7)	
		Carer	55	45 (81.8)	10 (18.2)

### Correlation coefficients between DASS-21 and SHS scores

**Fig. 3:**
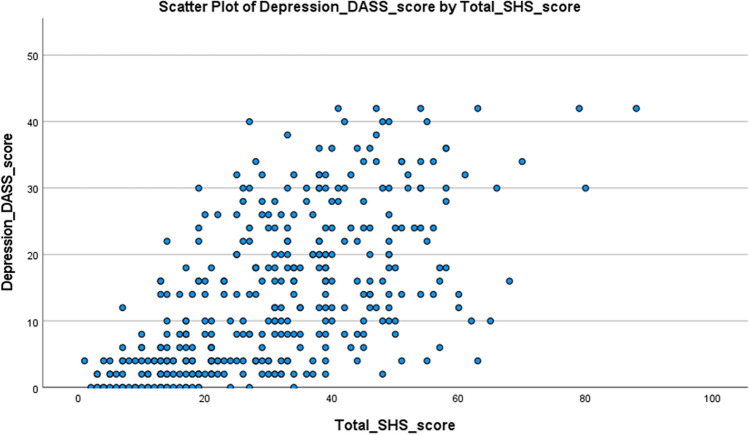
Scatterplot of depression score (DASS-21) versus total SHSQ-25 score

**Fig. 4:**
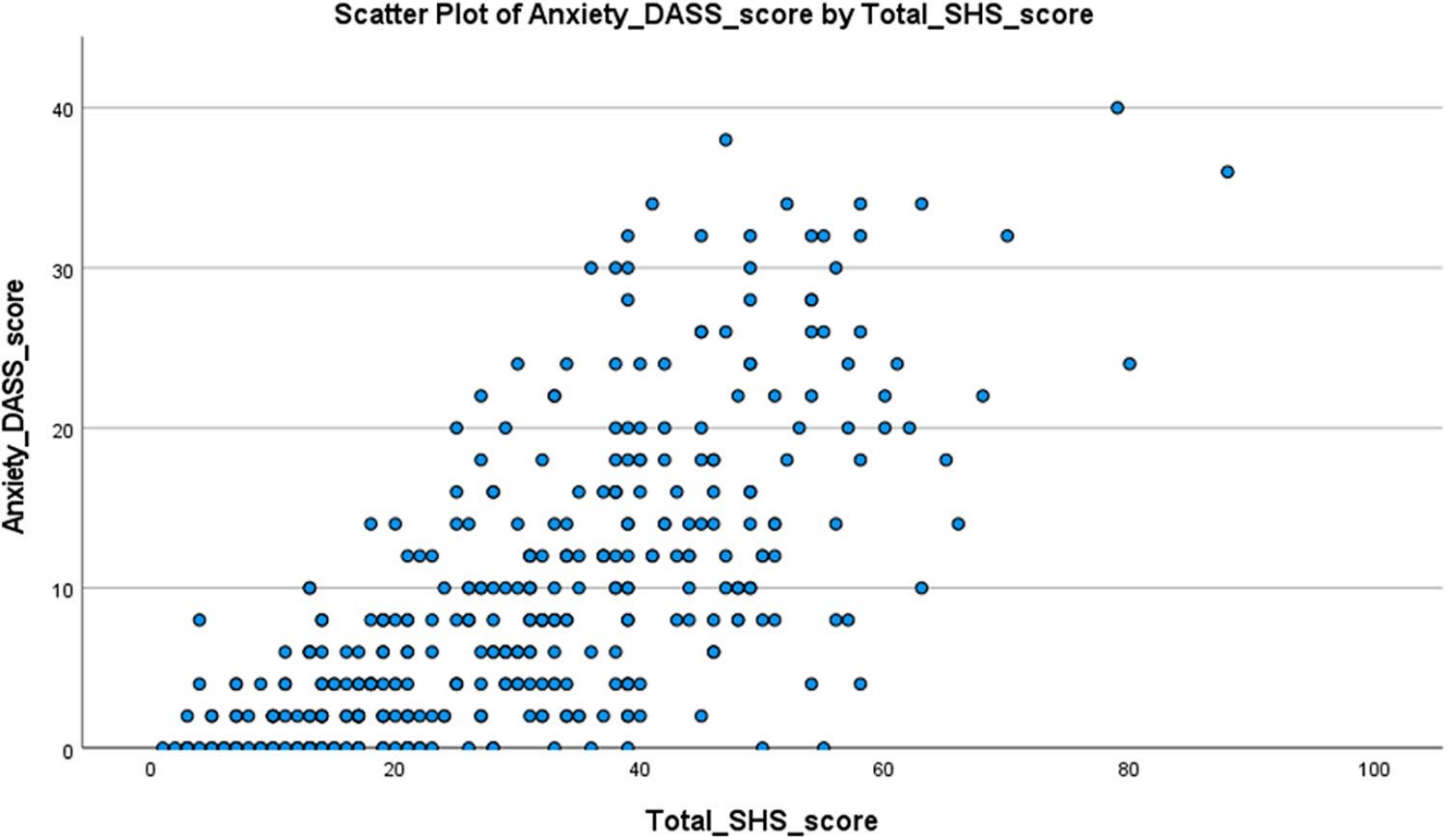
Scatterplot of anxiety score (DASS-21) versus total SHSQ-25 score

**Fig. 5:**
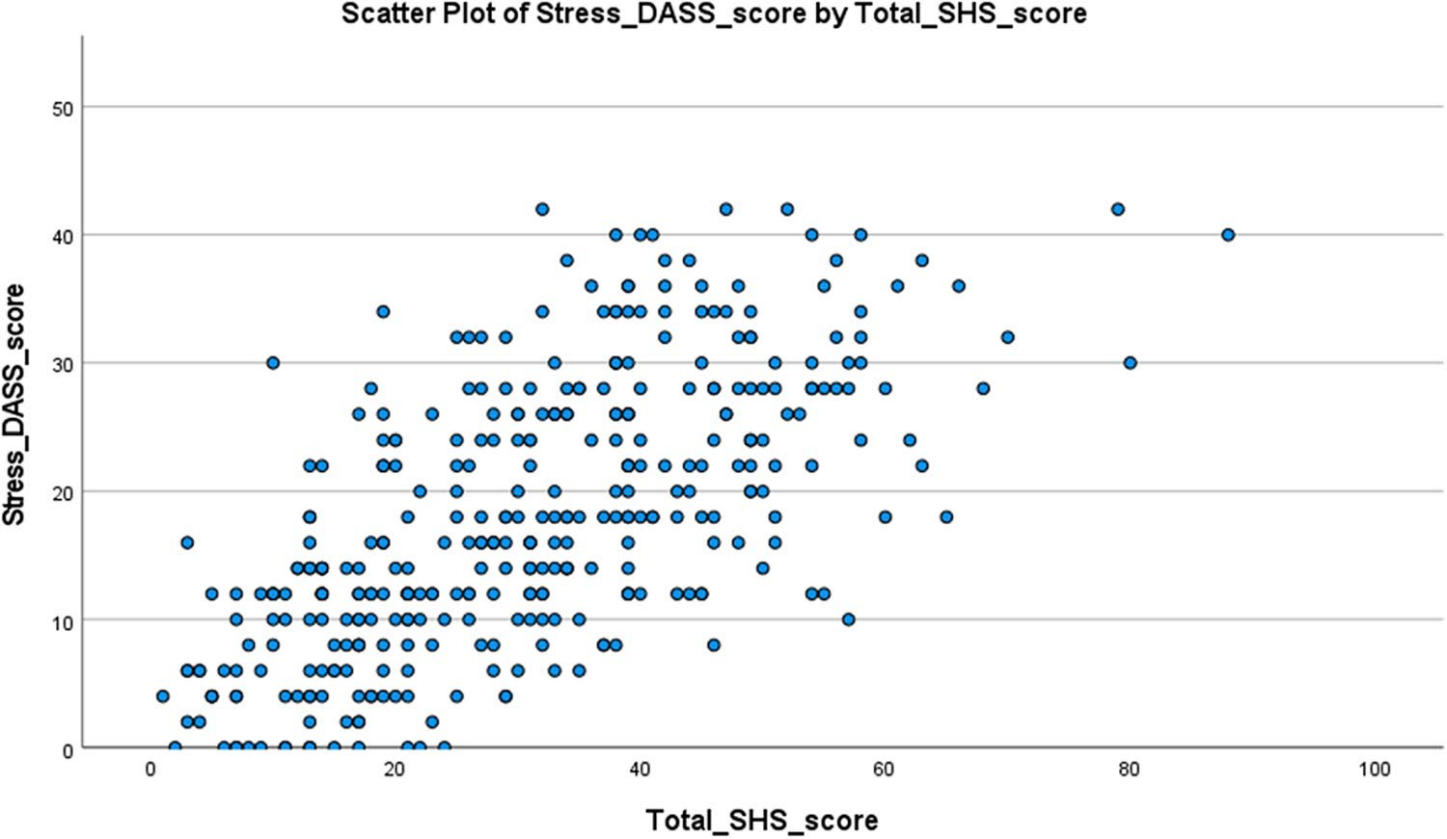
Scatterplot of stress score (DASS-21) versus total SHSQ-25 score

Bivariate correlation analysis between each dimension of the DASS-21 and SHS scores for the total population (*n* = 368) revealed positive correlations (Table 7). Depression scores and SHS scores for the total population (*n* = 368) revealed a moderate correlation, with the Spearman correlation coefficient (*r*) of 0.635, which was statistically significant (*p* < 0.001). Anxiety scores and SHS scores for the total population (*n* = 368) revealed a moderate correlation, with the Spearman correlation coefficient (*r*) of 0.713, which was statistically significant (*p* < 0.001). Stress scores and SHS scores for the total population (368) revealed a significant correlation, with the Spearman correlation coefficient (*r*) of 0.674 (*p* < 0.001) (Figs. 3, 4 and 5).

**Table 7:** Frequencies of participants with psychological symptoms

	Depression (+ve)	Depression (−ve)	Anxiety (+ve)	Anxiety (−ve)	Stress (+ve)	Stress (−ve)
Carer (SHS)	89	9	87	11	88	10
General population (SHS)	14	7	17	4	17	4
Carer (OH)	64	41	53	52	60	45
General population (OH)	39	105	35	109	39	105

### Logistic regression analysis on SHS between carers and the general population

Logistic regression analysis was performed to ascertain the effects of the caring role with SHS development. After adjusting for population type (carer vs general population), we found that carers were 6.4 times more likely to have SHS than the general population (aOR = 6.400, 95% CI = 3.751–10.919). The logistic regression model was statistically significant (*p* < 0.001) (Table 8).

**Table 8:** Multivariate logistic regression analysis for SHS

Variables	*B*	SE	Wald	*p*	aOR	95% CI
Population type (1)	1.856	0.273	46.383	*<0.001*	6.400	3.751–10.919

Population type: carers (1)

## Discussion

PPPM shifts the paradigm from reactive to proactive healthcare by providing a unique window of opportunity to prevent chronic disease [8]. Predictive medical approaches include, among other things, screening assessments to effectively predict individuals at risk of developing chronic diseases [7]. This study has demonstrated that the SHSQ-25 can accurately predict that carers are at far greater risk of developing chronic diseases than the general population, facilitating population level SHS prediction. This provides a unique window of opportunity for targeted population-level prevention. As SHS shares modifiable (behavioural) risk factors with chronic diseases, early identification will allow clinicians to prescribe personalised treatment algorithms [7]. Tailoring treatments to specific individuals will mitigate the development of chronic diseases in carers. Stratifying individuals into high (SHS) and low (optimal health) risk based on the SHS cut-off value could improve personalised medicine for chronic diseases from the perspective of PPPM.

Effective screening of chronic diseases, for example CVD and T2DM, is crucial in PPPM’s quest to mitigate chronic disease prevalence [15]. In line with the working hypothesis of PPPM, SHS is proactive in its approach by predicting a person’s predisposition to developing chronic diseases, creating a unique window of opportunity for the prevention and personalised treatment of chronic diseases, initiating the prescription of early interventions in at-risk individuals [3]. This simple, inexpensive and validated screening tool facilitates early identification of high-risk individuals, allowing for timely employment of targeted prevention and personalised treatment algorithms. This shared outcome by PPPM and SHS has the capacity to alter the course of disease trajectories, potentially preventing disease manifestations from occurring altogether [8]. These benefits extend beyond the state-of-the-art to improve quality of life outcomes and mitigate economic burden. At a time when global healthcare is overwhelmed by the perpetuity of chronic disease burden, SHS, with all its potential, should be adopted as the new gold standard in the fight against chronic diseases from the perspectives of PPPM.

For the first time to our knowledge, this study found unpaid, informal carers had a significantly higher SHS prevalence than in the general population (43.0% vs 12.7%), demonstrating carers are more susceptible to SHS than that in the general population in Australia. The median total SHS scores and IQR were statistically higher in carers compared to the general population (38.0; IQR 28.0–48.0 vs 20.0; IQR 13.0–32.0) as well as all domains, including fatigue (17.0; IQR 13.0~22.0 vs 9.0; IQR 6.0~14.0), cardiovascular (1.0; IQR 0.0–3.0 vs 1.0; IQR 0.0~1.0), digestive (3.0; IQR 1.0~4.0 vs 1.0; IQR 0.0~3.0), immune (2.0; IQR 1.0~4.0 vs 2.0; IQR 1.0–3.0) and mental health (12.0; IQR 9.0~17.0 vs 6.0; IQR 3.0~11.0), indicating carers suffer from poorer health than that in the general population. These findings mirrored psychological symptom prevalence. For example, we found that carers had a higher prevalence of depression than that in the general population (75.4% vs 32.1%), a higher prevalence of anxiety than the general population (69.0% vs 31.5%) and a higher prevalence of stress than the general population (72.9% vs 33.9%). Further, we found carers had significantly higher median depression, anxiety and stress scores than the general population, respectively (16.0; IQR 10.0~28.0 vs 4.0; IQR 2.0~12.0) (10.0; IQR 6.0~8.0 vs 4.0, IQR 0.0~8.0) (22.0; IQR 14.0~8.012.0; IQR 6.0~18.0), indicating carers suffered from more severe psychological symptoms than that in the general population. Our research shows, in the post-COVID-19 era, where unpaid carers have increased mental health conditions and are developing chronic diseases earlier, the value of identifying/predicting which carers via at risk of developing mental health conditions, and their sequelae, before their manifestation. This personalised approach, in the spirit of PPPM, allows optimal health care by predictive diagnosis and targeted prevention. In turn, this enables valuable resources to be directed to where they are most needed, with the aim of avoiding disease manifestation.

Psychological symptoms are known to contribute to the development of SHS. In this study, we explored the relationship between psychological symptoms and SHS prevalence in two populations in order to understand the influence of the caring role on SHS from the perspective of PPPM. We found that psychological symptoms were associated with SHS, and that carers with SHS experienced a higher prevalence of psychological symptoms than that in the general population with SHS. Further, SHS was significantly correlated with psychological symptoms with the Spearman correlation coefficient of 0.635 for depression, 0.713 for anxiety and 0.674 for stress symptoms, respectively. Further, the caregiving role was found to influence SHS prevalence, with carers 6.4 times more likely to suffer from SHS than the general population. This finding identifies the impact of the caregiving role on the health status in otherwise healthy carers and the urgent need under the concept of PPPM to proactively intervene in this vulnerable, disadvantaged population at a time when prevention of chronic diseases is still possible.

Against the concept of PPPM, the current biomedical model of health has been reactive in its response to managing chronic diseases, typically initiating treatments only after the clinical manifestation of disease [30]. The impact of chronic diseases is enormous, with an increase in total global costs predicted for cancer, CVD, chronic obstructive pulmonary disease, T2DM and mental illness, respectively [6]. SHS screening is proactive in its approach by predicting which individuals are at risk of developing certain chronic diseases before their onset, enabling the delivery of targeted, PPPM algorithms to prevent disease from occurring in the first place [8, 20]. From the lens of PPPM, this study found that the SHSQ-25 could accurately predict “at-risk” individuals for psychological and health problems. For example, within the general population, the median depression score among those with SHS was 12.0 (IQR 6.0–25.0), whilst the median depression score among those with optimal health was 4.0 (2.0–10.0), indicating SHS is associated with depressive symptoms. Similarly, the median anxiety score among those in the general population with SHS was 18.0 (9.0–32.0), whilst the median anxiety score among those with optimal health was 2.0 (0.0–6.0), indicating that SHS is associated with anxiety symptoms and optimal health is associated with no anxiety symptoms.

Stress is a key contributing factor in the development of SHS [8]. The human stress response is an evolutionarily conserved, dynamic process that aims to overcome a stressor (a real or perceived threat), promote survival and restore homeostasis [32]. The role of the two major components involved in the stress response—the sympatho-adrenomedullary (SAM) axis (producing catecholamines) and hypothalamic-pituitary-adrenal (HPA) axis (producing cortisol), respectively, is well documented [32, 33]. However, during times of chronic stress, that is, stress that persists over time, these processes become maladaptive [32]. In chronic stress, hypothalamic activation of the pituitary switches from CRH to vasopressin, decreasing the metabolism of cortisol [32]. Elevated cortisol levels and long-term cortisol exposure result, which are toxic to the human body and can lead to chronic disease [32]. It has been well-documented that carers experience substantial chronic stress as part of their caring role [8]. For example, a recent study by the Royal Australian College of General Practitioners found approximately 27% of primary carers had high psychological distress, almost three times higher than that in the general population [22]. Moreover, the carer well-being survey found that 48.1% of carers experienced moderate to high levels of psychological distress, almost twice as many as non-carers (25%) [27]. Our results were consistent with these findings, with the median depression score among carers with SHS being 22.0 (IQR 14.0~30.5), whilst the median depression score among the general population with SHS was 12.0 (IQR 6.0~25.0), indicating that carers with SHS suffered from more severe depression problems compared to the general population with SHS. Conversely, the median depression score among carers with optimal health was 14.0 (4.0–22.0) whereas the median depression score among the general population with optimal health was 4.0 (2.0–10.0), indicating that carers without SHS still experienced psychological symptoms, whilst the general population with optimal health experienced an absence of depressive symptoms. Further, the median anxiety score among carers with optimal health was 8.0 (4.0–12.0), whereas the median anxiety score among the general population with optimal health was 2.0 (0.0–6.0), indicating that carers without SHS still suffered from anxiety symptoms, whilst the general population with optimal health showed no anxiety symptoms.

From a personalised medicine approach, one of the three components of PPPM, our findings have particular relevance to women’s health. It is prudent to note that the overwhelming majority of participants in our study were females, with 93.1% of carers (*n* = 189) identifying as female and 87.9% of the general population (*n* = 145) identifying as female. We found female carers were more susceptible to SHS than females in the general population, with a significantly higher prevalence of 50.3% (95/189) compared to 12.4% (18/145). The medians and IQRs for total SHS scores were higher among female carers (39.0, IQR 29.0–48.5) than females in the general population (20.0, IQR 13.0–32.0) indicating female carers suffered more psychological problems than females in the general population. Depression scores were higher in female carers (18.0, IQR 10.0–28.0) than in females in the general population (4.0, IQR 2.0–12.0); anxiety scores were higher in female carers (10.0, IQR 6.0–28.0) than that of females in the general population (3.0, IQR 0.0–8.0); and stress scores were higher in female carers (22.0; IQR 14.0–28.0) than females in the general population (12.0, IQR 6.0–19.0). These findings hold great significance for women’s health considering anxiety and depression disorders were among the leading causes of total burden plaguing Australian females in 2022 [5]. For example, according to the Australian Institute of Health and Welfare’s Australian Burden of Disease Study 2022, anxiety disorders were the leading cause of total burden in females aged between 15 and 24 years, accounting for 14,300 individuals, or 10.2% of this age group [4]. Depressive disorders were the second cause of total burden, accounting for 11,400 individuals, or 8.2% [5]. In females aged between 25 and 44 years, anxiety disorders were again the leading cause of total burden, accounting for 39,300 individuals, or 8.9% of this age group [5]. Depressive disorders were the third cause of total burden, accounting for 32,600 individuals, or 7.4%. In females aged between 45 and 64 years, anxiety disorders were the fourth cause of total burden, accounting for 30,100 individuals, or 4.6% of this age group [5]. Depressive disorders were the fifth cause of total burden, accounting for 25,500 individuals, or 3.9% [5]. In our study, the majority of female carers were aged between 45 and 64 years (59.1%, *n* = 120) compared to 35.2% (*n* = 58) of the general population, with carers aged 25–44 years accounting for 36% (*n* = 73) compared to 53.9% (*n* = 89) of the general population.

The SHSQ-25 was able to accurately distinguish between females within the population who were at significantly greater risk of developing chronic diseases (in this study female carers). Importantly, these carers were otherwise healthy and free from disease. Further, this study found carers who were aged between 25 and 64 years had significantly higher DASS-21 scores than the general population (Table S6). These findings should serve to guide priority funding for future women’s health prevention strategies, especially considering primary prevention measures are proven to be highly cost-effective in healthcare practice following PPPM principles [10]. For example, an investment in collaborative care in primary care that addressed psychological stress and the risk of depression and anxiety disorders (that have been associated with T2DM and CVD) found the investment was cost saving, with a positive return on investment of $1.52 for every $1 invested after just 2 years [34]. Despite the growing body of evidence showing that millions of deaths can be avoided by focusing on prevention, with billions of dollars saved, investment in health promotion activities remains incredulously low, with only 2–4% of total health sector spending spent on prevention activities in most countries [35, 36]. Investment in the SHSQ-25 for the screening and detection of SHS in carers would offer a simple, low-cost, highly effective PPPM tool in the global fight against chronic diseases.

It is known that chronic diseases can manifest as a result of non-classical, modifiable risk factors (such as stress) and can induce behavioural changes such as poor diet, sedentary behaviour and substance abuse, and that this may adversely impact the health status of an individual [6]. Indeed, SHS has been shown not only to be associated with subjective markers (such as psychological symptoms) but has also been associated with objective biomarkers [8]. These include cardiovascular risk factors (including increased systolic and diastolic blood pressure, total cholesterol, HDL cholesterol, triglycerides and plasma glucose) [8], increased plasma cortisol levels and GRB/GRA mRNA ratio [8], and endothelial dysfunction [8]. This underscores the importance of interventions designed specifically for carers. This is especially so considering carers are more sedentary than the general population.

For example, recent research has shown that carers are less likely to participate in sufficient physical activity compared to the general population, with the authors surmising that the age of carers, or a carers own disability, may have played a role in this observation [37]. This lack of physical activity in carers compared to non-carers was consistent with our results, with three quarters of all carers (75.4%) doing no vigorous exercise each week, compared to just over half (53.9%) of the general population. Interestingly however, we can confirm that the age of carers, or their disabilities, was not contributing factor in our study. In our results, the median age of carers and the general population were strikingly similar (46 years v 40 years), with all participants enrolled in the study free from any disability or comorbidity. We therefore hypothesise that carers are less likely to participate in sufficient exercise due to the demands of the caring role and the psychological impact that that exerts on their capacity to engage in exercise. Recent research found monitoring of carer health and morbidity, particularly of “at-risk” individuals such as female carers with asthma or diabetes, remains important [34]. Indeed, we found carers who ate no or less fruit were more susceptible to SHS (62.2%; 23/37) than the general population who ate no fruit (31.8%; 7/22), and carers who did not participate in any mild or moderate exercise were more susceptible to SHS (54.3%; 44/81) than the general population (8.1%; 3/37).

Carers are more likely to experience significant health problems than non-carers in Australia [26] with the role associated with a higher mortality rate than the general population [27]. The most common reason for taking on the role of an informal carer is a sense of family responsibility (70.1%) [7]. More recently, researchers discovered that carers have a higher prevalence of chronic conditions than the general population and are able to elucidate the specific chronic diseases involved: mental health disorders, CVD, T2DM, chronic obstructive pulmonary disease, asthma and arthritis [38]. A follow-up study from the same authors found carers had a higher prevalence of depression, anxiety, T2DM and arthritis than the general population, were more likely to report their health as being fair/poor and were more likely to have higher BMI and waist-hip ratio (WHR) than the general population [37]. Further, research has found carers have different biomedical profiles compared to the general population [37].

This study demonstrates that screening carers via the SHSQ-25, through the lens of PPPM, affords a predictive approach by identifying firstly that carers are at greater risk of developing chronic disease than the general public.

Specifically, carers were found to have significantly lower serum vitamin D and haemoglobin levels than the general population. The vitamin D finding is significant considering its role in preventing osteoporosis, especially considering post-menopausal women make up more than half of all female carers [37]. Caregiver-based studies need to become a part of mainstream biomedical research at both epidemiological and clinical levels [38]. Moreover, research has shown that COVID-19 has dramatically increased the early development of chronic diseases in unpaid carers.

### Expert recommendation

Aspects presented in this article encompass the needs of unpaid carers, personalised screening assessments for SHS (via the SHSQ-25) to predict the risk of chronic disease development, and the cost-efficacy of implementing such a program in this vulnerable population. These pillars are in alignment with the paradigm change from reactive medicine to PPPM as promoted by the EPMA. Adoption of these recommendations has the capacity to decrease delayed diagnosis and intervention, reduce untargeted prevention and ineffective treatment, and ultimately improve quality of life outcomes for carers and healthcare system sustainability [7]. Recognising the SHSQ-25 and its role in screening for SHS will contribute to PPPM’s principles by going being the state of the art, shifting the paradigm from reactive to proactive medicine.

For the first time from the perspective of PPPM, we made the novel discovery that healthy carers, who are yet to experience significant health problems, are 6.4 times more likely to develop SHS than healthy members of the general population. We recommend:

1. Identification of SHS enables targeted interventions to be initiated before the onset of clinical symptoms, delaying or changing disease trajectories, and preventing disease from occurring in the first place in the primary care setting [8].
2. As a PPPM tool, SHS’s capacity to deliver personalised care directs valuable resources to where they are most needed, conferring both health and economic benefits to healthcare systems [8].
3. Considering there are over 860,000 primary carers in Australia, the majority being female, and the fact that they experience a higher prevalence of health problems compared to females in the general population, our results are a sobering reminder to governments of the disadvantage faced by carers in the social determinants of health.
4. Our research should serve to guide future policies and direct future women’s health prevention strategies. Investment in the SHSQ-25 for the screening of SHS in carers would offer a simple, low-cost, highly effective tool for the detection of SHS.
5. Effective PPPM measures, such as SHS, that can reduce chronic disease burden by the smallest proportion and have the capacity to significantly mitigate their economic impact on health systems, warrant special attention [4]. This is especially true considering the WHO’s recent calls for evidence-based, cost-effective disease prevention strategies to alleviate the burgeoning costs and complications of chronic diseases [10].

This research evidences the SHSQ-25 as the novel PPPM tool in the fight against chronic disease in women’s health, and more specifically, unpaid female carers.

## Limitations

This was a cross-sectional study, which meant we were unable to measure the causality of the caregiving role influences with SHS and psychological symptoms. Further, collected survey data was self-reported by participants, which may have introduced some degree of information bias.

## Conclusions

Innovative strategies within the working hypothesis of PPPM view SHS as a predictive medical approach with cost-effective preventive measures, affording a unique window of opportunity to prevent the development of chronic diseases before they manifest in the primary care setting. Our research shows that using the SHSQ-25 as a screening tool in unpaid carers effectively predicts which individuals are at risk of chronic disease development, providing timely intervention of personalised treatments, and preventing chronic disease from occurring in the first place.

For example, for the first time from the perspective of PPPM, this study found unpaid, informal carers had a significantly higher SHS prevalence than that in the general population (43.0% vs 12.7%), demonstrating carers are more susceptible to SHS than that in the general population in Australia. Further, we made the novel discovery that healthy carers, who are yet to experience significant health problems, are 6.4 times more likely to develop SHS than healthy members of the general population. Moreover, we found that psychological symptoms, including depression, anxiety and stress, were all associated with SHS, and that carers with SHS experienced a higher prevalence of mental health symptoms than those in the general population with SHS. These findings have particular relevance for women’s health, especially considering mental health disorders are among the leading causes of total burden for females in Australia.

## Supplementary Information

Below is the link to the electronic supplementary material.

Supplementary file1 (DOCX 120 KB)

## Data Availability

No datasets were generated or analysed during the current study.

## Abbreviations

SHS: Suboptimal Health Status
SHSQ-25: Suboptimal Health Status Questionnaire-25
WHO: World Health Organisation
DASS-21: Depression Anxiety Stress Scale-21
DALY: Disability-adjusted life years
PPPM: Predictive, preventive and personalised medicine
IQR: Interquartile ranges
SD: Standard deviation
SPSS: Statistical Package for the Social Sciences
ECU: Edith Cowan University
IACO: International Alliance of Carer Organisation
HREC: Human Research Ethics Committee
*X*^2^: Chi-squared test
IQR: Interquartile ranges
aOR: Adjusted odds ratio
CI: Confidence interval
*r*: Spearman correlation coefficient
