# Supplementary material for: The caregiving role influences Suboptimal Health Status and psychological symptoms in unpaid carers
Source: EPMA J. 2024 Jul 31;15(3):453–69. doi: 10.1007/s13167-024-00370-8 (PMC11372173; doi:10.1007/s13167-024-00370-8)
Supplement: Supplementary file 1 — Supplementary file1 (DOCX 120 KB) [file 13167_2024_370_MOESM1_ESM.docx]

|  |  |  |  |  |  |  |  |
| --- | --- | --- | --- | --- | --- | --- | --- |
|  |  |  |  | Table S1 SHS prevalence lifestyle |  |  |  |
|  |  |  |  |  |  |  |  |
|  | **Variables** |  | **Total (N=368)** | **Healthy (%) (N=249)** | **SHS (%) (N=119)** | **X²** | ***p* value** |
|  |  |  |  |  |  |  |  |
| Smoking  status | No | General population | 145 | 129 (89.0) | 16 (11.0) | 52.480 | ***<0.001*** |
|  |  | Carer | 176 | 90 (51.1) | 86 (48.9) |  |  |
|  | Yes | General population | 20 | 15 (75.0) | 5 (25.0) | 1.882 | ***<0.001*** |
|  |  | Carer | 27 | 15 (55.6) | 12 (44.4) |  |  |
| Alcohol  consumption | Once a week | General population | 60 | 53 (88.3) | 7 (11.7) | 15.134 | ***<0.001*** |
|  |  | Carer | 68 | 39 (57.4) | 29 (42.6) |  |  |
|  | Once a fortnight | General population | 13 | 11 (84.6) | 2 (15.4) | 6.667 | ***0.01*** |
|  |  | Carer | 14 | 5 (35.7) | 9 (64.3) |  |  |
|  | Once a month | General population | 48 | 41 (84.5) | 7 (84.6) | 12.412 | ***<0.001*** |
|  |  | Carer | 48 | 25 (52.1) | 23 (47.9) |  |  |
|  | Never | General population | 44 | 39 (88.6) | 5 (11.4) | 18.446 | ***<0.001*** |
|  |  | Carer | 73 | 36 (49.3) | 37 (50.7) |  |  |
| Caffeine | 0 | General population | 20 | 18 (90.0) | 2 (10.0) | 10.193 | ***0.001*** |
|  |  | Carer | 23 | 10 (43.5) | 13 (56.5) |  |  |
|  | 1 | General population | 25 | 22 (88.0) | 3 (12.0) | 9.687 | ***0.002*** |
|  |  | Carer | 31 | 15 (48.4) | 16 (51.6) |  |  |
|  | 2 | General population | 72 | 63 (87.5) | 9 (12.5) | 10.077 | ***0.002*** |
|  |  | Carer | 55 | 35 (63.6) | 20 (36.4) |  |  |
|  | 3 | General population | 29 | 25 (86.2) | 4 (13.8) | 11.477 | ***0.001*** |
|  |  | Carer | 39 | 18 (46.2) | 21 (53.8) |  |  |
|  | 4 or more | General population | 19 | 16 (84.2) | 3 (15.8) | 7.155 | ***0.007*** |
|  |  | Carer | 55 | 27 (49.1) | 28 (50.9) |  |  |
| Sedentary  behaviour | Mostly sitting | General population | 106 | 92 (86.8) | 14 (20.9) | 28.226 | ***<0.001*** |
|  |  | Carer | 115 | 62 (53.9) | 53 (34.9) |  |  |
|  | Mostly standing | General population | 27 | 25 (92.6) | 2 (7.4) | 15.427 | ***<0.001*** |
|  |  | Carer | 45 | 21 (46.7) | 24 (53.3) |  |  |
|  | Mostly walking | General population | 20 | 18 (90.0) | 2 (10.0) | 4.749 | ***0.029*** |
|  |  | Carer | 26 | 16 (61.5) | 10 (38.5) |  |  |
|  | Mostly doing heavy labour | General population | 12 | 9 (75.0) | 3 (25.0) | 4.441 | ***0.035*** |
|  |  | Carer | 17 | 6 (35.3) | 11 (64.7) |  |  |

|  |  | Table S2 SHS prevalence social | |  |  | |  |  |  |  |
| --- | --- | --- | --- | --- | --- | --- | --- | --- | --- | --- |
|  |  |  | |  |  | |  |  |  |  |
|  | **Variables** |  | **Total (N=368)** | | | **Healthy (%) (N=249)** | | **SHS (%) (N=119)** | **X²** | **p value** |
|  |  |  |  | | |  | |  |  |  |
| Attendance of community events | Once a week or more | General population | 30 | | | 29 (96.7) | | 1 (3.3) | 8.953 | ***0.005*** |
|  |  | Carer | 20 | | | 13 (65.0) | | 7 (35.0) |  |  |
|  | Once a fortnight | General population | 15 | | | 12 (80.0) | | 3 (20.0) | 0.077 | 1.000 |
|  |  | Carer | 8 | | | 6 (75.0) | | 2 (20.0) |  |  |
|  | Once a month | General population | 56 | | | 51 (91.1) | | 5 (8.9) | 11.059 | ***0.002*** |
|  |  | Carer | 25 | | | 15 (60.0) | | 10 (40.0) |  |  |
|  | Once every couple of months | General population | 22 | | | 20 (90.9) | | 2 (9.1) | 6.111 | ***0.013*** |
|  |  | Carer | 33 | | | 20 (60.6) | | 13 (39.4) |  |  |
|  | A few times a year | General population | 22 | | | 18 (81.8) | | 4 (18.2) | 8.797 | ***0.003*** |
|  |  | Carer | 68 | | | 31 (45.6) | | 37 (54.4) |  |  |
|  | Never | General population | 20 | | | 14 (70.0) | | 6 (30.0) | 4.840 | ***0.028*** |
|  |  | Carer | 49 | | | 20 (49.3) | | 29 (50.7) |  |  |
| Do you have a pet/s | No | General population | 54 | | | 49 (90.7) | | 5 (9.3) | 19.953 | ***<0.001*** |
|  |  | Carer | 54 | | | 28 (51.9) | | 26 (48.1) |  |  |
|  | Yes | General population | 111 | | | 95 (85.6) | | 16 (14.4%) | 32.664 | ***<0.001*** |
|  |  | Carer | 149 | | | 77 (51.7) | | 72 (48.3) |  |  |
| How often do you go into nature | Once a week | General population | 113 | | | 103 (91.2) | | 10 (8.8) | 19.407 | ***<0.001*** |
|  |  | Carer | 75 | | | 49 (65.3) | | 26 (34.7) |  |  |
|  | Once a fortnight | General population | 18 | | | 15 (83.3) | | 3 (16.7) | 7.946 | ***0.005*** |
|  |  | Carer | 33 | | | 14 (42.4) | | 19 (57.6) |  |  |
|  | Once a month | General population | 14 | | | 11 (78.6) | | 3 (21.4) | 3.913 | ***0.048*** |
|  |  | Carer | 26 | | | 12 (46.2) | | 14 (53.8) |  |  |
|  | Once every couple of months | General population | 20 | | | 15 (75.0) | | 5 (25.0) | 6.163 | ***0.013*** |
|  |  | Carer | 69 | | | 30 (43.5) | | 39 (56.5) |  |  |
| Meeting with family | Once a week or more | General population | 62 | | | 58 (93.5) | | 4 (6.5) | 22.289 | ***<0.001*** |
|  |  | Carer | 26 | | | 13 (50.0) | | 13 (50.0) |  |  |
|  | Once a fortnight | General population | 39 | | | 32 (82.1) | | 7 (17.9) | 1.708 | 0.191 |
|  |  | Carer | 32 | | | 22 (68.8) | | 10 (31.2) |  |  |
|  | Once a month | General population | 17 | | | 15 (88.2) | | 2 (11.8) | 2.080 | 0.269 |
|  |  | Carer | 26 | | | 18 (69.2) | | 8 (30.8) |  |  |
|  | Once every couple of months | General population | 17 | | | 15 (88.2) | | 2 (11.8) | 14.306 | ***<0.001*** |
|  |  | Carer | 51 | | | 18 (35.3) | | 33 (65.7) |  |  |
|  | A few times a year | General population | 16 | | | 14 (87.5) | | 2 (12.5) | 5.664 | ***0.017*** |
|  |  | Carer | 41 | | | 22 (53.7) | | 19 (46.3) |  |  |
|  | Never | General population | 14 | | | 10 (71.4) | | 4 (18.6) | 2.700 | 0.100 |
|  |  | Carer | 27 | | | 12 (44.4) | | 15 (55.6) |  |  |
| Meeting with friends | Once a week or more | General population | 43 | | | 38 (88.4) | | 5 (11.6) | 4.900 | ***0.041*** |
|  |  | Carer | 13 | | | 8 (61.5) | | 5 (38.5) |  |  |
|  | Once a fortnight | General population | 24 | | | 23 (95.8) | | 1 (4.2) | 7.639 | ***0.011*** |
|  |  | Carer | 25 | | | 16 (64.0) | | 9 (36.0) |  |  |
|  | Once a month | General population | 54 | | | 45 (83.3) | | 9 (16.7) | 7.396 | ***0.007*** |
|  |  | Carer | 35 | | | 20 (57.1) | | 15 (42.9) |  |  |
|  | Once every couple of months | General population | 18 | | | 17 (94.4) | | 1 (5.6) | 12.262 | ***<0.001*** |
|  |  | Carer | 45 | | | 21 (46.7) | | 24 (53.3) |  |  |
|  | A few times a year | General population | 17 | | | 15 (88.2) | | 2 (11.8) | 5.493 | ***0.019*** |
|  |  | Carer | 46 | | | 26 (56.5) | | 20 (43.5) |  |  |
|  | Never | General population | 9 | | | 6 (66.7) | | 3 (33.3) | 2.848 | 0.137 |
|  |  | Carer | 39 | | | 14 (35.9) | | 25 (64.1) |  |  |
| Large gatherings | Once a week or more | General population | 1 | | | 1(100.0) | | 0 (0) | 3.000 | 0.333 |
|  |  | Carer | 2 | | | 0 (0) | | 2 (100.0) |  |  |
|  | Once a fortnight | General population | 8 | | | 7 (87.5) | | 1 (12.5) | 0.141 | 1.000 |
|  |  | Carer | 1 | | | 1 (100.0) | | 0 (0) |  |  |
|  | Once a month | General population | 9 | | | 9 (100.0) | | 0 (0) | 2.438 | 0.308 |
|  |  | Carer | 4 | | | 3 (75.0) | | 1 (25.0) |  |  |
|  | Once every couple of months | General population | 31 | | | 29 (93.5) | | 2 (6.5) | 9.948 | ***0.004*** |
|  |  | Carer | 21 | | | 12 (57.1) | | 9 (42.9) |  |  |
|  | A few times a year | General population | 78 | | | 66 (84.6) | | 12 (15.4) | 14.813 | ***<0.001*** |
|  |  | Carer | 65 | | | 36 (55.4) | | 29 (44.6) |  |  |
|  | Never | General population | 38 | | | 32 (84.2) | | 6 (15.8) | 14.996 | ***<0.001*** |
|  |  | Carer | 110 | | | 53 (48.2) | | 57 (51.8) |  |  |
| Social groups | No | General population | 102 | | | 85 (83.3) | | 17 (16.7) | 35.668 | ***<0.001*** |
|  |  | Carer | 152 | | | 70 (46.1) | | 82 (53.9) |  |  |
|  | Yes | General population | 63 | | | 59 (93.7) | | 4 (6.3) | 12.200 | ***<0.001*** |
|  |  | Carer | 51 | | | 35 (68.6) | | 16 (31.4) |  |  |

|  |  |  |  | Table S3 SHS prevalence diet |  |  |  |
| --- | --- | --- | --- | --- | --- | --- | --- |
|  |  |  |  |  |  |  |  |
|  |  |  |  |  |  |  |  |
|  | **Variables** |  | **Total(N=368)** | **Healthy (%) (N=249)** | **SHS (%) (N=119)** | **X²** | **p Value** |
|  |  |  |  |  |  |  |  |
| Fruits (serves) | 0 | General population | 22 | 15 (68.2) | 7 (31.8) | 5.083 | ***0.024*** |
|  |  | Carer | 37 | 14 (37.8) | 23 (62.2) |  |  |
|  | 1 | General population | 89 | 79 (88.8) | 10 (11.2) | 33.300 | ***<0.001*** |
|  |  | Carer | 92 | 45 (48.9) | 47 (51.1) |  |  |
|  | 2 | General population | 37 | 34 (91.9) | 3 (8.1) | 11.018 | ***0.001*** |
|  |  | Carer | 56 | 34 (60.7) | 22 (39.3) |  |  |
|  | 3 or more | General population | 17 | 16 (94.1) | 1 (5.9) | 4.118 | 0.088 |
|  |  | Carer | 18 | 12 (66.7) | 6 (33.3) |  |  |
| Vegetables (serves) | 0 | General population | 3 | 0 (0) | 3 (100.0) | 1.518 | 0.515 |
|  |  | Carer | 14 | 5 (35.7) | 9 (64.3) |  |  |
|  | 1 | General population | 39 | 32 (82.1) | 7 (17.9) | 11.184 | ***0.001*** |
|  |  | Carer | 56 | 27 (48.2) | 29 (51.8) |  |  |
|  | 2 | General population | 66 | 59 (89.4) | 7 (10.6) | 26.294 | ***<0.001*** |
|  |  | Carer | 63 | 30 (47.6) | 33 (52.4) |  |  |
|  | 3 | General population | 32 | 30 (93.8) | 2 (6.3) | 12.201 | ***<0.001*** |
|  |  | Carer | 45 | 26 (57.8) | 19 (42.2) |  |  |
|  | 4 | General population | 15 | 14 (93.3) | 1 (6.7) | 2.706 | 0.178 |
|  |  | Carer | 17 | 12 (70.6) | 5 (29.4) |  |  |
|  | More than 5 | General population | 10 | 9 (90.0) | 1 (10.0) | 1.945 | 0.275 |
|  |  | Carer | 8 | 5 (62.5) | 3 (37.5) |  |  |
| Takeaway foods | Daily | General population | 1 | 0 (0) | 1 (100.0) | 1.875 | 0.400 |
|  |  | Carer | 4 | 3 (75.0) | 1 (25.0) |  |  |
|  | 2-6 times a week | General population | 30 | 25 (83.3) | 5 (16.7) | 17.160 | ***<0.001*** |
|  |  | Carer | 25 | 7 (28.0) | 18 (72.0) |  |  |
|  | Once a week | General population | 37 | 32 (86.5) | 5 (13.5) | 11.689 | ***0.001*** |
|  |  | Carer | 47 | 24 (51.1) | 23 (48.9) |  |  |
|  | Once a fortnight | General population | 45 | 40 (88.9) | 5 (11.1) | 15.920 | ***<0.001*** |
|  |  | Carer | 44 | 22 (50.0) | 22 (50.0) |  |  |
|  | Once a month or less | General population | 52 | 47 (90.4) | 5 (9.6) | 15.294 | ***<0.001*** |
|  |  | Carer | 83 | 49 (59.0) | 34 (41.0) |  |  |
| Fried foods | Daily | General population | 4 | 1 (25.0) | 3 (75.0) | 0.000 | 1.000 |
|  |  | Carer | 4 | 1 (25.0) | 3 (75.0) |  |  |
|  | 2-6 times a week | General population | 34 | 30 (88.2) | 4 (11.8) | 12.854 | ***<0.001*** |
|  |  | Carer | 39 | 19 (48.7) | 20 (51.3) |  |  |
|  | Once a week | General population | 51 | 42 (82.4) | 9 (17.6) | 15.216 | ***<0.001*** |
|  |  | Carer | 49 | 22 (44.9) | 27 (55.1) |  |  |
|  | Once a fortnight | General population | 36 | 34 (94.4) | 2 (5.6) | 17.190 | ***<0.001*** |
|  |  | Carer | 44 | 23 (52.3) | 21 (47.7) |  |  |
|  | Once a month or less | General population | 40 | 37 (92.5) | 3 (7.5) | 13.354 | ***<0.001*** |
|  |  | Carer | 67 | 40 (59.7) | 27 (40.3) |  |  |
| Sugary drinks | Daily | General population | 23 | 16 (69.6) | 7 (30.4) | 4.719 | ***0.030*** |
|  |  | Carer | 39 | 16( 41.0) | 23 (59.0) |  |  |
|  | 2-6 times a week | General population | 32 | 25 (78.1) | 7 (21.9) | 4.605 | ***0.032*** |
|  |  | Carer | 34 | 18 (52.9) | 16 (47.1) |  |  |
|  | Once a week | General population | 17 | 16 (94.1) | 1 (5.9) | 11.836 | ***0.001*** |
|  |  | Carer | 18 | 7 (38.9) | 11 (61.1) |  |  |
|  | Once a fortnight | General population | 26 | 22 (84.6) | 4 (15.4) | 10.889 | ***0.001*** |
|  |  | Carer | 21 | 8 (38.1) | 13 (61.9) |  |  |
|  | Once a month or less | General population | 67 | 65 (97.0) | 2 (3.0) | 27.081 | ***<0.001*** |
|  |  | Carer | 91 | 56 (61.5) | 35 (38.5) |  |  |
| Sweet foods | Daily | General population | 39 | 33 (84.6) | 6 (15.4) | 11.987 | ***0.001*** |
|  |  | Carer | 56 | 28 (50.0) | 28 (50.0) |  |  |
|  | 2-6 times a week | General population | 80 | 69 (86.3) | 11 (13.7) | 31.754 | ***<0.001*** |
|  |  | Carer | 97 | 44 (45.4) | 53 (54.6) |  |  |
|  | Once a week | General population | 32 | 28 (87.5) | 4 (12.5) | 1.125 | 0.319 |
|  |  | Carer | 26 | 20 (76.9) | 6 (23.1) |  |  |
|  | Once a fortnight | General population | 5 | 5 (100.0) | 0 (0.00) | 5.078 | 0.075 |
|  |  | Carer | 8 | 3 (37.5) | 5 (62.5) |  |  |
|  | Once a month or less | General population | 9 | 9(100) | 0(0) | 4.441 | 0.057 |
|  |  | Carer | 16 | 10 (62.5) | 6 (37.5) |  |  |
| Salty snacks | Daily | General population | 6 | 3 (50.0) | 3 (50.0) | 0.000 | 1.000 |
|  |  | Carer | 8 | 4 (50.0) | 4 (50.0) |  |  |
|  | 2-6 times a week | General population | 41 | 33 (80.5) | 8 (19.5) | 8.603 | ***0.003*** |
|  |  | Carer | 51 | 26 (51.0) | 25 (49.0) |  |  |
|  | Once a week | General population | 44 | 39 (88.6) | 5 (11.4) | 19.067 | ***<0.001*** |
|  |  | Carer | 52 | 24 (46.2) | 28 (53.8) |  |  |
|  | Once a fortnight | General population | 34 | 30 (88.2) | 4 (11.8) | 10.783 | ***0.001*** |
|  |  | Carer | 33 | 17 (51.5) | 16 (48.5) |  |  |
|  | Once a month or less | General population | 40 | 39 (97.5) | 1 (2.5) | 19.571 | ***<0.001*** |
|  |  | Carer | 59 | 34 (57.6) | 25 (42.4) |  |  |
| Processed meat | Daily | General population | 4 | 1 (25.0) | 3 (75.0) | 0.533 | 1.000 |
|  |  | Carer | 4 | 2 (50.0) | 2 (50.0) |  |  |
|  | 2-6 times a week | General population | 26 | 23 (88.5) | 3 (11.5) | 11.664 | ***0.001*** |
|  |  | Carer | 57 | 28 (49.1) | 29 (50.9) |  |  |
|  | Once a week | General population | 61 | 52 (85.2) | 9 (14.8) | 16.375 | ***<0.001*** |
|  |  | Carer | 49 | 24 (49.0) | 25 (51.0) |  |  |
|  | Once a fortnight | General population | 30 | 28 (93.3) | 2 (6.7) | 18.276 | ***<0.001*** |
|  |  | Carer | 31 | 13 (41.9) | 18 (58.1) |  |  |
|  | Once a month or less | General population | 44 | 40 (90.9) | 4 (9.1) | 11.615 | ***0.001*** |
|  |  | Carer | 62 | 38 (61.2) | 24 (38.7) |  |  |
| Red meat | Daily | General population | 5 | 3 (60.0) | 2 (40.0) | 0.026 | 1.000 |
|  |  | Carer | 9 | 5 (55.6) | 4 (44.4) |  |  |
|  | 2-6 times a week | General population | 102 | 90 (88.2) | 12 (11.8) | 37.722 | ***<0.001*** |
|  |  | Carer | 123 | 61 (49.6) | 62 (50.4) |  |  |
|  | Once a week | General population | 28 | 27 (96.4) | 1 (3.6) | 16.065 | ***<0.001*** |
|  |  | Carer | 34 | 17 (50.0) | 17 (50.0) |  |  |
|  | Once a fortnight | General population | 9 | 9 (100.0) | 0 (0.0) | 3.706 | 0.104 |
|  |  | Carer | 12 | 8 (66.7) | 4 (33.3) |  |  |
|  | Once a month or less | General population | 21 | 15 (71.4) | 6 (28.6) | 1.166 | 0.280 |
|  |  | Carer | 25 | 14 (56.0) | 11 (44.0) |  |  |
| White meat | Daily | General population | 7 | 5 (71.4) | 2 (28.6) | 0.833 | 0.633 |
|  |  | Carer | 12 | 6 (50.0) | 6 (50.0) |  |  |
|  | 2-6 times a week | General population | 131 | 115 (87.8) | 16 (12.2) | 40.404 | ***<0.001*** |
|  |  | Carer | 145 | 76 (52.4) | 69 (47.6) |  |  |
|  | Once a week | General population | 12 | 11 (91.7) | 1 (8.3) | 6.969 | ***0.011*** |
|  |  | Carer | 20 | 9 (45.0) | 11 (55.0) |  |  |
|  | Once a fortnight | General population | 2 | 2 (100.0) | 0 (0.0) | 0.381 | 0.537 |
|  |  | Carer | 6 | 5 (83.3) | 1 (16.7) |  |  |
|  | Once a month or less | General population | 13 | 11 (84.6) | 2 (15.4) | 5.179 | ***0.023*** |
|  |  | Carer | 20 | 9 (45.0) | 11 (55.0) |  |  |

|  | **Variables** | Table S4 SHS prevalence exercise | **Total (N=368)** | **Healthy (%) (N=249)** | **SHS (%) (N=119)** | **X2** | **p value** |
| --- | --- | --- | --- | --- | --- | --- | --- |
| Mild/moderate exercise | None | General population | 37 | 34 (91.9) | 3 (8.1) | 22.633 | ***<0.001*** |
|  |  | Carer | 81 | 37 (45.7) | 44 (54.3) |  |  |
|  | 1-2 times | General population | 74 | 63 (85.1) | 11 (14.9) | 21.817 | ***<0.001*** |
|  |  | Carer | 77 | 38 (49.4) | 39 (50.6) |  |  |
|  | 3-5 times | General population | 41 | 37 (90.2) | 4 (9.8) | 5.357 | ***0.021*** |
|  |  | Carer | 32 | 22 (68.8) | 10 (31.3) |  |  |
|  | 5 times or more | General population | 13 | 10 (76.9) | 3 (23.1) | 0.722 | 0.673 |
|  |  | Carer | 13 | 8 (61.5) | 5 (38.5) |  |  |
| Time spent exercising | 1 hour or less | General population | 60 | 53 (88.3) | 7( 11.7) | 36.852 | ***<0.001*** |
|  |  | Carer | 116 | 47 (40.5) | 69 (59.5) |  |  |
|  | 2 hours | General population | 50 | 43 (86.0) | 7 (14.0) | 4.843 | ***0.028*** |
|  |  | Carer | 42 | 28 (66.7) | 14 (33.3) |  |  |
|  | 3 hours | General population | 21 | 18 (85.7) | 3 (14.3) | 1.542 | 0.254 |
|  |  | Carer | 16 | 11 (68.8) | 5 (31.2) |  |  |
|  | 4 hours | General population | 12 | 10 (83.3) | 2 (16.7) | 2.081 | 0.216 |
|  |  | Carer | 14 | 8 (57.1) | 6 (42.9) |  |  |
|  | 5 hours or more | General population | 22 | 20 (90.9) | 2 (9.1) | 2.028 | 0.198 |
|  |  | Carer | 15 | 11 (73.3) | 4 (26.7) |  |  |
| Vigorous exercise | None | General population | 89 | 75 (84.3) | 14 (15.7) | 33.708 | ***<0.001*** |
|  |  | Carer | 153 | 71 (46.4) | 82 (53.6) |  |  |
|  | 1-2 times | General population | 54 | 48 (88.9) | 6 (11.1) | 5.673 | ***0.017*** |
|  |  | Carer | 35 | 24 (68.6) | 1 1(31.4) |  |  |
|  | 3-5 times | General population | 17 | 17 (100.0) | 0 (0.0) | 7.983 | ***0.012*** |
|  |  | Carer | 10 | 6 (60.0) | 4 (40.0) |  |  |
|  | 5 times or more | General population | 5 | 4 (80.0) | 1 (20.0) | 0.000 | 1.000 |
|  |  | Carer | 5 | 4 (80.0) | 1 (20.0) |  |  |
| Time spent exercising | 1 hour or less | General population | 103 | 87 (84.5) | 16 (15.5) | 35.923 | ***<0.001*** |
|  |  | Carer | 163 | 78 (47.9) | 85 (52.1) |  |  |
|  | 2 hours | General population | 30 | 27 (90.0) | 3 (10.0) | 7.556 | ***0.006*** |
|  |  | Carer | 18 | 10 (55.6) | 8 (44.4) |  |  |
|  | 3 hours | General population | 16 | 16 (100.0) | 0 (0.0) | 1.852 | 0.360 |
|  |  | Carer | 9 | 8 (88.9) | 1 (11.1) |  |  |
|  | 4 hours | General population | 7 | 7 (100.0) | 0 (0.0) | 3.360 | 0.152 |
|  |  | Carer | 5 | 3 (60.0) | 2 (40.0) |  |  |
|  | 5 hours or more | General population | 9 | 7 (77.8) | 2 (22.2) | 0.018 | 1.000 |
|  |  | Carer | 8 | 6 (75.0) | 2 (25.0) |  |  |

|  |  |  |  |  | Table S5 SHS and domain scores demographics | | | | |
| --- | --- | --- | --- | --- | --- | --- | --- | --- | --- |
|  |  |  |  |  |  | | | | |
|  |  |  |  |  |  | | | | |
|  |  |  |  |  | **Domains of the SHSQ-25** | | | | |
| **Variables** |  |  | **N** | **Total Score** | **Fatigue** | **Mental health** | **Cardiovascular** | **Digestive** | **Immune** |
|  |  |  |  |  |  |  |  |  |  |
| Gender | Male | General population | 19 | 20.0 (9.0-33.0) | 9.0 (3.0-11.0) | 7.0 (3.0-14.0) | 0.0 (0.0-1.0) | 1.0 (0.0-3.0) | 2.0 (1.0-4.0) |
|  |  | Carer | 13 | 21.0 (15.5-36.0) | 13.0 (6.5-18.0) | 8.0 (4.5-12.0) | 0.0 (0.0-1.5) | 2.0 (1.0-3.0) | 1.0 (0.0-2.0) |
|  | Female | General population | 145 | 20.0 (13.0-32.0) | 9.0 (6.0-14.0) | 6.0 (3.0-11.0) | 1.0 (0.0-1.0) | 1.0 (0.0-3.0) | 2.0 (1.0-3.0) |
|  |  | Carer | 189 | 39.0 (29.0-48.5)* | 17.0 (13.0-22.0) | 13.0 (9.0-17.0) | 1.0 (0.0-3.0) | 3.0 (1.0-4.0) | 2.0 (1.0-4.0) |
|  | Nonbinary | General population | 1 | 39.0 (39.0-39.0) | 16.0 (16.0-16.0) | 19.0 (19.0-19.0) | 1.0 (1.0-1.0) | 1.0 (1.0-1.0) | 2.0 (2.0-2.0) |
|  |  | Carer | 1 | 62.0 (62.0-62.0) | 28.0 (28.0-28.0) | 12.0 (12.0-12.0) | 3.0 (3.0-3.0) | 10.0 (10.0-10.0) | 9.0 (9.0-9.0) |
| Age | 18-24 years | General population | 16 | 33.5 (15.5-38.7) | 13.0 (6.25-15.7) | 12.5 (5.5-18.5) | 0.0 (0.0-1.7) | 2.0 (1.0-3.0) | 2.5 (2.0-4.7) |
|  |  | Carer | 2 | 25.0 (24.0-25.0) | 12.5 (12.0-12.5) | 6.5 (6.0-6.5) | 1.5 (0.0-1.5) | 1.5 (1.0-1.5) | 3.0 (3.0-3.0) |
|  | 25-44 years | General population | 89 | 19.0 (13.0-32.5) | 9.0 (6.0-14.0) | 6.0 (2.5-12.0) | 0.0 (0.0-1.0) | 1.0 (0.0-2.5) | 2.0 (1.0-3.0) |
|  |  | Carer | 73 | 39.0 (29.0-48.5)* | 17.0 (13.0-22.0) | 14.0 (9.5-17.0) | 1.0 (0.0-2.5) | 3.0 (2.0-4.0) | 2.0 (1.0-4.5) |
|  | 45-64 years | General population | 58 | 20.0 (11.5-27.2) | 9.0 (6.0-14.0) | 6.0 (4.0-8.0) | 1.0 (0.0-1.0) | 1.0 (0.0-2.2) | 1.0 (0.0-30) |
|  |  | Carer | 120 | 38.0 (28.2-48.7)* | 17.0 (13.0-22.0) | 12.0 (8.2-17.0) | 1.0 (0.0-3.0) | 3.0 (1.0-4.0) | 2.0 (1.0-4.0) |
|  | 65 plus years | General population | 2 | 14.5 (11.0-14.5) | 5.0 (4.0-5.0) | 4.0 (3.0-4.0) | 1.0 (1.0-1.0) | 2.5 (1.0-2.5) | 2.0 (0.0-2.0) |
|  |  | Carer | 8 | 34.5 (30.7-38.7)* | 17.0 (14.0-19.0) | 11.0 (9.0-13.5) | 1.5 (1.0-2.0) | 3.0 (1.2-3.0) | 2.0 (1.2-3.0) |
| Marital status | Never married | General population | 57 | 26.0 (14.5-35.0) | 10.0 (7.0-16.5) | 8.0 (4.0-13.5) | 1.0 (0.0-2.0) | 2.0 (0.5-3.0) | 2.0 (1.0-3.0) |
|  |  | Carer | 32 | 38.5 (27.2-47.5)* | 17.0 (13.2-21.0) | 13.0 (11.0-17.0) | 1.0 (0.0-3.0) | 3.0 (2.0-4.0) | 2.0 (1.0-3.0) |
|  | Married | General population | 79 | 19.0 (11.0-27.0) | 8.0 (6.0-13.0) | 5.0 (3.0-8.0) | 1.0 (0.0-1.0) | 1.0 (0.0-2.0) | 2.0 (1.0-3.0) |
|  |  | Carer | 130 | 34.0 (25.7-46.0)* | 17.0 (12.7-21.0) | 12.0 (8.0-16.0) | 1.0 (0.0-3.0) | 2.0 (1.0-4.0) | 2.0 (1.0-5.0) |
|  | Widowed | General population | 5 | 19.0 (10.0-32.5) | 10.0 (3.50-12.5) | 6.0 (3.5-12.5) | 0.0 (0.0-2.0) | 1.0 (0.0-4.5) | 1.0 (0.5-4.0) |
|  |  | Carer | 3 | 40.0 (33.0-40.0) | 16.0 (15.0-16.0) | 11.0 (10.0-11.0) | 2.0 (2.0-2.0) | 4.0 (1.0-4.0) | 3.0 (0.0-3.0) |
|  | Divorced/separated | General population | 24 | 18.5 (10.2-32.0) | 7.5 (4.0-16.7) | 6.0 (3.0-9.0) | 0.5 (0.0-1.0) | 1.0 (0.0-4.7) | 1.0 (0.0-3.0) |
|  |  | Carer | 38 | 43.0 (33.7-54.5)* | 19.0 (15.7-26.0) | 14.0 (10.7-20.0) | 2.0 (1.0-4.0) | 3.0 (2.0-5.0) | 3.0 (1.0-4.0) |
| Education | Primary school | General population | 1 | 37.0 (37.0-37.0) | 15.0 (15.0-15.0) | 14.0 (14.0-14.0) | 0.0 (0.0-0.0) | 4.0 (4.0-4.0) | 4.0 (4.0-4.0) |
|  |  | Carer | 2 | 31.0 (31.0-31.0) | 13.5 (11.0-13.5) | 13.0 (12.0-13.0) | 0.0 (0.0-0.0) | 2.0 (1.0-2.0) | 2.5 (2.0-2.5) |
|  | High school | General population | 25 | 31.0 (17.5-39.0) | 13.0 (8.5-18.0) | 10.0 (6.0-15.0) | 1.0 (0.0-3.0) | 2.0 (1.0-3.0) | 2.0 (1.0-5.0) |
|  |  | Carer | 36 | 34.5 (26.5-46.7) | 17.0 (13.0-20.0) | 12.0 (10.2-15.0) | 1.0 (0.0-3.0) | 3.0 (2.0-4.0) | 1.5 (1.0-4.0) |
|  | TAFE/trade | General population | 46 | 19.5 (12.5-28.0) | 9.0 (6.0-12.5) | 6.0 (3.7-10.0) | 1.0 (0.0-1.0) | 2.0 (0.0-3.0) | 1.5 (1.0-3.0) |
|  |  | Carer | 75 | 43.0 (37.0-54.0)* | 20.0 (16.0-24.0) | 15.0 (11.0-18.0) | 1.0 (0.0-4.0) | 4.0 (2.0-5.0) | 3.0 (1.0-4.0) |
|  | Bachelor's degree | General population | 54 | 17.0 (10.0-32.2) | 8.0 (4.0-13.2) | 5.5 (2.0-9.5) | 0.0 (0.0-1.0) | 1.0 (0.0-2.2) | 1.0 (0.0-3.0) |
|  |  | Carer | 64 | 33.0 (26.0-44.7)* | 17.0 (12.0-19.7) | 11.0 (7.2-16.7) | 1.0 (0.0-3.0) | 2.0 (1.0-3.0) | 2.0 (1.0-3.7) |
|  | Postgraduate | General population | 39 | 19.0 (12.0-28.0) | 9.0 (6.0-15.0) | 5.0 (3.0-8.0) | 0.0 (0.0 -1.0) | 1.0 (0.0-2.0) | 2.0 (1.0-2.0) |
|  |  | Carer | 26 | 29.5 (18.0-40.5)* | 13.5 (8.7-18.5) | 9.0 (6.7-11.2) | 1.0 (0.0-2.0) | 2.0 (1.0-3.0) | 2.0 (1.0-4.2) |
| Income | Under $50k | General population | 23 | 30.0 (17.0-39.0) | 13.0 (6.0-18.0) | 9.0 (3.0-17.0) | 0.0 (0.0-2.0) | 1.0 (1.0-3.0) | 2.0 (2.0-4.0) |
|  |  | Carer | 76 | 39.5 (31.0-47.0)* | 18.5 (15.0-22.7) | 13.0 (10.0-18.0) | 1.0 (0.2-3.0) | 3.0 (2.0-4.0) | 2.0 (1.0-4.0) |
|  | $50-$69K | General population | 28 | 23.5 (14.2-35.5) | 10.0 (7.2-15.5) | 7.5 (4.2-13.7) | 0.5 (0.0-1.0) | 2.0 (0.2-4.0) | 2.0 (1.0-4.0) |
|  |  | Carer | 30 | 38.5 (27.7-49.2)* | 18.0 (14.0-23.0) | 12.5 (8.7-16.0) | 2.0 (0.0-3.0) | 3.0 (2.0-4.0) | 3.0 (1.0-5.0) |
|  | $70-$89k | General population | 27 | 21.0 (11.0-28.0) | 9.0 (5.0-13.0) | 6.0 (5.0-10.0) | 1.0 (0.0-1.0) | 1.0 (0.0-3.0) | 2.0 (1.0-3.0) |
|  |  | Carer | 32 | 31.5 (26.0-46.7)* | 17.0 (10.2-22.0) | 11.5 (8.0-16.0) | 1.0 (0.0-3.0) | 3.0 (1.0-4.0) | 2.5 (1.0-4.0) |
|  | $90-$109k | General population | 22 | 18.5 (12.5-21.5) | 9.0 (6.0-12.0) | 5.0 (2.0-7.0) | 1.0 (0.0-1.0) | 1.0 (0.0-2.0) | 1.0 (0.0-2.2) |
|  |  | Carer | 23 | 34.0 (25.0-50.0)* | 17.0 (13.0-21.0) | 13.0 (9.0-17.0) | 1.0 (0.0-2.0) | 2.0 (1.0-3.0) | 2.0 (1.0-3.0) |
|  | Over $110k | General population | 65 | 17.0 (11.0-30.0) | 8.0 (6.0-13.5) | 5.0 (3.0-11.0) | 1.0 (0.0-1.0) | 1.0 (0.0-2.0) | 1.0 (1.0 -2.5) |
|  |  | Carer | 42 | 32.5 (18.0-46.5)* | 15.0 (10.7-20.2) | 11.0 (7.0-15.2) | 1.0 (0.0-3.0) | 2.0 (1.0-4.0) | 2.0 (1.0-6.0) |
| Housing | Private rental | General population | 59 | 23.0 (16.0-34.0) | 10.0 (6.0-17.0) | 7.00 (3.0-12.0) | 0.0 (0.0-1.0) | 2.0 (0.0-3.0) | 2.0 (1.0-3.0) |
|  |  | Carer | 53 | 40.0 (30.5-51.0)* | 19.0 (13.0-22.5) | 15.0 (9.0-20.0) | 2.0 (2.0-4.0) | 3.0 (2.0-5.0) | 2.0 (1.0-4.0) |
|  | Public housing | General population | 3 | 22.0 (4.0-22.0) | 6.0 (2.0-6.0) | 7.0 (1.0-7.0) | 1.0 (0.0-1.0) | 1.0 (0.0-1.0) | 2.0 (1.0-2.0) |
|  |  | Carer | 9 | 39.0 (29.5-49.0) | 19.0 (14.0-21.5) | 14.0 (11.5-18.5) | 1.0 (0.0-2.5) | 3.0 (3.0-5.0) | 2.0 (0.5-3.5) |
|  | Being paid off | General population | 82 | 19.0 (11.7-31.0) | 9.0 (6.0-14.0) | 6.0 (3.0-11.0) | 1.0 (0.0-1.0) | 1.0 (0.0-2.2) | 1.0 (0.7-3.0) |
|  |  | Carer | 87 | 37.0 (27.0-49.0)* | 17.0 (13.0-21.0) | 13.0 (9.0-17.0) | 1.0 (0.0-3.0) | 2.0 (1.0-4.0) | 3. 0 (1.0-4.0) |
|  | Fully owned | General population | 21 | 15.0 (10.5-22.0) | 7.0 (4.0-9.5) | 5.0 (4.0-6.5) | 0.0 (0.0-1.0) | 1.0 (0.0-2.0) | 1.0 (0.0-4.0) |
|  |  | Carer | 54 | 34.5 (28.5-40.5)* | 17.0 (12.7-22.0) | 10.5 (7.0-14.0) | 1.0 (0.0-3.0) | 3.0 (1.0-4.0) | 2.0 (1.0-4.2) |
| Living arrangements | Living alone | General population | 29 | 21.0 (15.0-35.5) | 9.0 (4.5-17.0) | 7.0 (4.0-12.0) | 1.0 (0.0-1.0) | 2.0 (0.0-4.0) | 2.0 (1.0-4.0) |
|  |  | Carer | 27 | 46.0 (31.0-54.0)* | 19.0 (16.0-22.0) | 17.0 (11.0-20.0) | 2.0 (0.0-4.0) | 3.0 (1.0-5.0) | 2.0 (1.0-4.0) |
|  | Living with partner | General population | 103 | 19.0 (12.0-29.0) | 9.0 (6.0-14.0) | 5.0 (3.0-9.0) | 1.0 (0.0-1.0) | 1.0 (0.0-2.0) | 2.0 (1.0-3.0) |
|  |  | Carer | 134 | 34.0 (25.0-46.0)* | 17.0 (12.0-21.0) | 12.0 (7.7-16.0) | 1.0 (0.0-3.0) | 2.0 (1.0-4.0) | 2.0 (1.0-4.0) |
|  | Living with parents | General population | 12 | 28.0 (14.7-38.0) | 10.5 (7.0-14.0) | 9.5 (4.2-15.0) | 1.0 (0.0-2.7) | 1.5 (1.0-2.7) | 3.0 (1.0-5.7) |
|  |  | Carer | 15 | 39.0 (31.0-44.0)* | 19.0 (16.0-23.0) | 11.0 (9.0-15.0) | 1.0 (0.0-2.0) | 3.0 (2.0-4.0) | 2.0 (1.0-5.0) |
|  | Living with other family/friends | General population | 21 | 19.0 (11.5-34.5) | 9.0 (4.0-14.0) | 7.0 (3.5-14.0) | 0.0 (0.0-1.0) | 2.0 (0.0-3.0) | 1.0 (0.0-2.0) |
|  |  | Carer | 27 | 41.0 (31.0-51.0)* | 19.0 (13.0-24.0) | 12.0 (11.0-19.0) | 2.0 (0.0-3.0) | 3.0 (2.0-5.0) | 3.0 (2.0-5.0) |
| Private health | No | General population | 57 | 23.0 (13.5-36.0) | 9.0 (6.0-15.0) | 8.0 (5.0-13.0) | 1.0 (0.0-2.0) | 2.0 (0.0-4.0) | 2.0 (1.0-4.0) |
|  |  | Carer | 93 | 41.0 (31.0-51.5)* | 19.0 (15.0-23.0) | 14.0 (10.0-19.0) | 2.0 (0.0-4.0) | 3.0 (2.0-5.0) | 3.0 (1.0-4.0) |
|  | Yes | General population | 108 | 19.0 (12.0 -28.0) | 9.0 (6.0-14.0) | 5.5 (3.0-8.7) | 0.0 (0.0-1.0) | 1.0 (0.0-2.0) | 1.0 (1.0-3.0) |
|  |  | Carer | 110 | 33.0 (25.7-44.2)* | 16.0 (12.0-20.0) | 11.0 (8.0-15.0) | 1.0 (0.0-2.0) | 2.0 (1.0-4.0) | 2.0 (1.0-4.0) |
| Pension card | No | General population | 139 | 19.0 (13.0-29.0) | 9.0 (6.0-14.0) | 6.0 (3.0-9.0) | 1.0 (0.0-1.0) | 1.0 (0.0-3.0) | 2.0 (1.0-3.0) |
|  |  | Carer | 102 | 32.5 (25.0-46.0)* | 16.0 (12.0-21.0) | 11.0 (7.7-16.0) | 1.0 (0.0-2.2) | 2.0 (1.0-4.0) | 2.0 (1.0-4.0) |
|  | Yes | General population | 26 | 29.5 (16.2-36.5) | 12.0 (6.0-18.2) | 10.5 (6.0-14.2) | 0.5 (0.0-3.0) | 1.0 (1.0-3.5) | 2.0 (1.0-4.0) |
|  |  | Carer | 101 | 40.0 (32.5-49.0)* | 19.0 (16.0-23.0) | 14.0 (10.0-18.0) | 2.0 (0.0-3.0) | 3.0 (2.0-4.5) | 2.0 (1.0-4.0) |
| Locality | Metro | General population | 144 | 20.0 (13.0-32.0) | 9.0 (6.0-14.7) | 6.0 (3.0-11.0) | 1.0 (0.0-1.0) | 1.0 (0.0-3.0) | 2.0 (1.0-3.0) |
|  |  | Carer | 160 | 39.0 (29.0-49.0)* | 17.0 (13.0-22.0) | 13.0 (9.0-17.0) | 1.0 (0.0-3.0) | 3.0 (1.0-4.0) | 2.0 (1.0-4.0) |
|  | Rural | General population | 18 | 19.5 (12.7-29.7) | 9.0 (7.5-10.2) | 5.0 (2.7-9.0) | 0.5 (0.0-2.0) | 2.0 (0.0-3.2) | 1.0 (0.7-3.2) |
|  |  | Carer | 42 | 33.5 (27.5-43.0)* | 17.0 (12.7-21.0) | 11.0 (8.0-15.2) | 1.0 (0.0-2.0) | 3.0 (1.0-3.0) | 2.0 (1.0-4.2) |
|  | Remote | General population | 3 | 22.0 (7.0-22.0) | 6.0 (5.0-6.0) | 7.0 (0.0-7.0) | 1.0 (1.0-1.0) | 2.0 (0.0-2.0) | 3.0 (1.0-3.0) |
|  |  | Carer | 1 | 30.0 (30.0-30.0) | 11.0 (11.0-11.0) | 11.0 (11.0-11.0) | 0.0 (0.0-0.0) | 5.0 (5.0-5.0) | 3.0 (3.0-3.0) |
| Dependents | No | General population | 72 | 19.5 (13.0-33.0) | 9.0 (6.0-14.0) | 7.0 (4.0-11.0) | 1.0 (0.0-1.0) | 1.0 (0.0-3.0) | 2.0 (1.0-3.0) |
|  |  | Carer | 86 | 34.0 (26.7-44.2)* | 17.0 (13.0-21.0) | 11.0 (7.7-15.2) | 1.0 (0.0-3.0) | 3.0 (1.0-4.0) | 2.0 (1.0-3.0) |
|  | Yes | General population | 93 | 20.0 (11.5-30.5) | 10.0 (6.0-14.0) | 6.0 (3.0-11.0) | 1.0 (0.0-1.0) | 1.0 (0.0-2.0) | 2.0 (1.0-3.0) |
|  |  | Carer | 117 | 39.0 (30.0-50.0)* | 18.0 (13.0-22.0) | 14.0 (10.0-18.0) | 1.0 (0.0-3.0) | 3.0 (1.0-4.0) | 3.0 (1.0-5.0) |
| Overall |  | General population | 165 | 20.0 (13.0-32.0) | 9.0 (6.0-14.0) | 1.0 (0.0-1.0) | 1.0 (0.0-3.0) | 2.0 (1.0-3.0) | 6.0 (3.0-11.0) |
|  |  | Carer | 203 | 38.0 (28.0-38.0) | 17.0 (13.0-22.0) | 1.0 (0.0-3.0) | 3.0 (1.0-4.0) | 2.0 (1.0-4.0) | 12.0 (9.0-17.0) |

**p*=0.05 for statistical tests

|  |  | Table S6 DASS scores demographics |  |  | | |
| --- | --- | --- | --- | --- | --- | --- |
|  |  |  |  |  | | |
|  |  |  |  |  | | |
|  |  |  |  | **Dimensions of the DASS-21** | | |
| **Variables** |  |  | **N** | **Depression score** | **Anxiety score** | **Stress score** |
|  |  |  |  |  |  |  |
| Gender | Male | General population | 19 | 4.0 (0.0-16.0) | 2.0 (0.0-6.0) | 6.0 (4.0-14.0) |
|  |  | Carer | 13 | 16.0 (8.0-18.0)* | 8.0 (3.0-11.0)* | 18.0 (10.0-27.0)* |
|  | Female | General population | 145 | 4.0 (2.0-12.0) | 4.0 (0.00-8.00) | 12.0 (6.0-19.0) |
|  |  | Carer | 189 | 18.0 (10.0-28.0)* | 10.0 (6.0-18.0)* | 22.0 (14.0-28.0)* |
|  | Nonbinary | General population | 1 | 32.0 (32.0-32.0) | 32.0 (32.-32.0) | 30.0 (30.0-30.0) |
| Age | 18-24 years | General population | 16 | 12.0 (4.0-20.0) | 6.0 (2.0-27.0) | 15.0 (3.5-29.5) |
|  |  | Carer | 2 | 7.0 (4.0-7.0) | 8.0 (2.0-8.0) | 16.0 (10.0-16.0) |
|  | 25-44 years | General population | 89 | 6.0 (2.0-17.0) | 4.0 (0.0-10.0) | 12.0 (6.0-21.0) |
|  |  | Carer | 73 | 16.0 (8.0-27.0)* | 12.0 (6.0-18.0)* | 22.0 (16.0-28.0)* |
|  | 45-64 years | General population | 58 | 4.0 (2.0-8.0) | 2.0 (0.0-6.0) | 9.0 (4.0-14.0) |
|  |  | Carer | 120 | 18.0 (10.0-28.0)* | 10.0 (6.0-18.0)* | 22.0 (14.0-28.0)* |
|  | 65 and over years | General population | 2 | 3.0 (2.0-3.0) | 5.0 (4.0-5.0) | 8.0 (4.0-8.0) |
|  |  | Carer | 8 | 13.0 (8.5-19.5) | 9.0 (4.5-11.5) | 15.0 (10.5-22.5) |
| Marital status | Never married | General population | 57 | 10.0 (4.0-20.0) | 4.0 (2.0-14.0) | 14.0 (9.0-23.0) |
|  |  | Carer | 32 | 23.0 (10.5-34.0)* | 10.0 (8.0-19.5)* | 22.0 (16.5-28.0)* |
|  | Married | General population | 79 | 4.0 (0.0-8.0) | 2.0 (0.0-6.0) | 10.0 (4.0-16.0) |
|  |  | Carer | 130 | 16.0 (8.0-22.0)* | 10.0 (4.0-16.0)* | 22.0 (12.0-28.0)* |
|  | Widowed | General population | 5 | 12.0 (2.0-22.0) | 8.0 (1.0-13.0) | 8.0 (0.0-28.0) |
|  |  | Carer | 3 | 30.0 (28.0-30.0) | 18.0 (4.0-18.0) | 26.0 (22.0-26.0) |
|  | Divorced/separated | General population | 24 | 4.0 (2.0-7.5) | 4.0 (0.0-6.0) | 11.0 (4.0-16.0) |
|  |  | Carer | 38 | 24.0 (10.0-32.0)* | 12.0 (6.0-22.0)* | 21.0 (16.0-30.5)* |
| Education | Primary school | General population | 1 | 20.0 (20.0-20.0) | 2.0 (2.0-2.0) | 8.0 (8.0-8.0) |
|  |  | Carer | 2 | 10.0 (2.0-10.0) | 7.0 (6.0-7.0) | 11.0 (10.0-11.0) |
|  | High school | General population | 25 | 10.0 (3.0-20.0) | 8.0 (4.0-21.0) | 16.0 (9.0-25.0) |
|  |  | Carer | 36 | 24.0 (12.5-31.5)* | 10.0 (4.5-18.0) | 26.0 (14.0-29.5) |
|  | TAFE/trade | General population | 46 | 6.0 (2.0-16.5) | 4.0 (2.0-8.0) | 12.0 (6.0-24.0) |
|  |  | Carer | 75 | 20.0 (14.0-30.0)* | 14.0 (8.0-22.0)* | 24.0 (16.0-32.0)* |
|  | Bachelor's degree | General population | 54 | 4.0 (2.0-12.0) | 4.0 (0.0-8.0) | 12.0 (4.0-16.5) |
|  |  | Carer | 64 | 15.0 (8.0-22.0)* | 10.0 (4.5-16.0)* | 18.0 (14.0-26.0)* |
|  | Postgraduate | General population | 39 | 4.0 (2.0-6.0) | 2.0 (0.0-4.0) | 10.0 (4.0-16.0) |
|  |  | Carer | 26 | 12.0 (7.5-16.0)* | 9.0 (4.0-15.0)* | 21.0 (12.0-26.0)* |
| Income | Under $50k | General population | 23 | 12.0 (4.0-20.0) | 6.0 (2.0-14.0) | 16.0 (8.0-28.0) |
|  |  | Carer | 76 | 22.0 (10.5-32.0)* | 12.0 (8.0-18.0)* | 24.0 (16.0-28.0) |
|  | $50-$69K | General population | 28 | 6.0 (2.0-17.0) | 4.0 (2.0-19.0) | 14.0 (4.5-28.0) |
|  |  | Carer | 30 | 16.0 (11.5-28.5)* | 12.0 (4.0-18.5)* | 25.0 (17.5-30.0)* |
|  | $70-$89k | General population | 27 | 4.0 (2.0-12.0) | 4.0 (2.0-6.0) | 12.0 (4.0-16.0) |
|  |  | Carer | 32 | 16.0 (12.0-25.5)* | 11.0 (4.5-17.5)* | 18.0 (14.0-26.0)* |
|  | $90-$109k | General population | 22 | 4.0 (1.5-8.5) | 3.0 (0.0-8.0) | 12.0 (4.0-14.5) |
|  |  | Carer | 23 | 14.0 (8.0-20.0)* | 8.0 (6.0-12.0)* | 20.0 (16.0-26.0)* |
|  | Over $110k | General population | 65 | 4.0 (2.0-8.0) | 2.0 (0.0-8.0) | 10.0 (6.0-17.0) |
|  |  | Carer | 42 | 10.0 (4.0-22.5)* | 8.0 (4.0-16.5)* | 22.0 (12.0-28.5)* |
| Housing | Renting from a real estate agency | General population | 59 | 6.0 (2.0-18.0) | 4.0 (0.0-10.0) | 12.0 (6.0-22.0) |
|  |  | Carer | 53 | 18.0 (10.0-30.0)* | 12.0 (8.0-21.0)* | 24.0 (16.0-31.0)* |
|  | Public housing | General population | 3 | 4.0 (4.0-4.0) | 8.0 (2.0-8.0) | 10.0 (2.0-10.0) |
|  |  | Carer | 9 | 30.0 (15.0-33.0) | 10.0 (8.0-21.0) | 20.0 (14.0-32.0) |
|  | Being paid off by you/ your partner | General population | 82 | 4.0 (2.0-12.0) | 4.0 (2.0-8.0) | 12.0 (6.0-18.0) |
|  |  | Carer | 87 | 16.0 (10.0-24.0)* | 10.0 (6.0-18.0)* | 22.0 (14.0-28.0)* |
|  | Fully owned/outright owned | General population | 21 | 2.0 (1.0-7.0) | 4.0 (0.0-8.0) | 8.0 (4.0-13.0) |
|  |  | Carer | 54 | 15.0 (8.0-24.5)* | 10.0 (4.0-14.0)* | 22.0 (12.0-26.0)* |
| Living arrangements | Living alone | General population | 29 | 6.0 (3.0-13.0) | 4.0 (0.0-8.0) | 10.0 (4.0-16.0) |
|  |  | Carer | 27 | 28.0 (18.0-36.0)* | 18.0 (8.0-26.0)* | 24.0 (18.0-30.0)* |
|  | Living with my partner | General population | 103 | 4.0 (2.0-8.0) | 2.0 (0.0-8.0) | 12.0 (6.0-16.0) |
|  |  | Carer | 134 | 16.0 (8.0-22.0)* | 10.0 (4.0-16.0)* | 22.0 (14.0-28.0)* |
|  | Living with my parents | General population | 12 | 14.0 (2.0-19.5) | 9.0 (4.0-24.5) | 19.0 (12.5-26.5) |
|  |  | Carer | 15 | 28.0 (8.0-34.0) | 10.0 (8.0-20.0) | 28.0 (14.0-30.0) |
|  | Living with other family/friends | General population | 21 | 10.0 (2.0-19.0) | 4.0 (0.0-17.0) | 14.0 (7.0-27.0) |
|  |  | Carer | 27 | 18.0 (10.0-30.0)* | 12.0 (6.0-16.0)* | 22.0 (12.0-28.0) |
| Private health | No | General population | 57 | 4.0 (2.0-16.0) | 4.0 (2.0-12.0) | 12.0 (6.0-23.0) |
|  |  | Carer | 93 | 20.0 (12.0-30.0)* | 12.0 (8.0-20.0)* | 24.0 (15.0-30.0)* |
|  | Yes | General population | 108 | 4.0 (2.0-10.0) | 4.0 (0.00-8.0) | 12.0 (4.0-18.0) |
|  |  | Carer | 110 | 14.0 (8.0-24.0)* | 9.0 (4.0-16.0)* | 22.0 (14.0-26.0)* |
| Pension card | No | General population | 139 | 4.0 (2.0-10.0) | 2.0 (0.0-8.0) | 12.0 (4.0-16.0) |
|  |  | Carer | 102 | 14.0 (7.5-24.0)* | 10.0 (6.0-14.5)* | 22.0 (13.5-26.5)* |
|  | Yes | General population | 26 | 12.0 (4.0-22.0) | 7.0 (1.5-14.5) | 19.0 (11.5-28.5) |
|  |  | Carer | 101 | 20.0 (12.0-30.0)* | 12.0 (6.0-18.0)* | 24.0 (15.0-30.0) |
| Locality | Metro | General population | 144 | 4.0 (2.0-12.0) | 4.0 (0.0-8.0) | 12.0 (6.0-18.0) |
|  |  | Carer | 160 | 18.0 (10.0-26.0)* | 12.0 (6.0-18.0)* | 22.0 (14.-28.00)* |
|  | Rural | General population | 18 | 5.0 (3.5-16.0) | 3.0 (1.5-12.5) | 12.0 (6.0-27.5) |
|  |  | Carer | 42 | 16.0 (7.5-32.0)* | 10.0 (4.0-16.0) | 21.0 (16.0-26.0)* |
|  | Remote | General population | 3 | 4.0 (0.0-4.0) | 4.0 (2.0-4.0) | 10.0 (4.0-10.0) |
|  |  | Carer | 1 | 4.0 (4.0-4.0) | 6.0 (6.0-6.0) | 18.0 (18.0-18.0) |
| Dependent children | No | General population | 72 | 4.0 (2.5-13.5) | 4.0 (0.0-8.0) | 12.0 (4.0-18.0) |
|  |  | Carer | 86 | 18.0 (10.0-28.0)* | 10.0 (6.0-16.5)* | 22.0 (14.0-28.0)* |
|  | Yes | General population | 93 | 4.0 (2.0-12.0) | 4.0 (1.0-9.0) | 12.0 (6.0-19.0) |
|  |  | Carer | 117 | 16.0 (8.0-26.0)* | 10.0 (6.0-18.0)* | 22.0 (14.0-29.0)* |

**p*=0.05 for statistical tests
